# SHIP blocks (MDP + LPS)-induced synergy in macrophages independent of catalytic activity

**DOI:** 10.1093/jleuko/qiag090

**Published:** 2026-06-27

**Authors:** Yvonne C F Pang, Susan C Menzies, Laura M Sly

**Affiliations:** Department of Pediatrics, Division of Gastroenterology, BC Children's Hospital and the University of British Columbia, Room A5-142, BC Children's Hospital Research Institute, 950 West 28th Avenue, Vancouver, BC V5Z 4H4, Canada; Department of Pediatrics, Division of Gastroenterology, BC Children's Hospital and the University of British Columbia, Room A5-142, BC Children's Hospital Research Institute, 950 West 28th Avenue, Vancouver, BC V5Z 4H4, Canada; Department of Pediatrics, Division of Gastroenterology, BC Children's Hospital and the University of British Columbia, Room A5-142, BC Children's Hospital Research Institute, 950 West 28th Avenue, Vancouver, BC V5Z 4H4, Canada

**Keywords:** IL-1β, NOD2, SHIP, TLR4

## Abstract

Macrophages coordinate cytokine responses by integrating signals from pattern recognition receptors including NOD2 and TLR4. NOD2 detects muramyl dipeptide derived from bacterial peptidoglycan, and TLR4 recognizes lipopolysaccharide in the outer membrane of Gram-negative bacteria. NOD2 and TLR4 signals synergize to enhance cytokine production in myeloid cells. While synergy can support host defense, it must be regulated to minimize the risk of excessive inflammation. The lipid phosphatase SHIP, a negative regulator of Class I PI3Ks, reduces inflammatory signaling, but its role during NOD2-TLR4 co-stimulation remains undefined. We found that SHIP limits IL-1β production by blocking synergy in bone marrow-derived macrophages co-stimulated with muramyl dipeptide and lipopolysaccharide. SHIP−/− macrophages showed a synergistic increase in IL-1β that was not evident in SHIP+/+ macrophages. Moreover, reducing SHIP protein concentrations by differentiation in different growth factors, IL-4 treatment, or siRNAs enabled synergy for IL-1β production in SHIP+/+ macrophages. Pharmacologic inhibition of SHIP's catalytic activity did not promote synergy, and similarly, blocking PI3K had no effect, suggesting that the response is independent of SHIP's phosphatase activity. Synergy for IL-1β production was dependent on NOD2 signaling despite NOD2 stimulation alone resulting in little to no IL-1β. Moreover, IL-1β was selectively enhanced in SHIP−/− macrophages during co-stimulation with MDP and LPS, or when MDP stimulation preceded LPS. These findings identify SHIP's adaptor function as a negative regulator of IL-1β induced by NOD2-TLR4 co-stimulation in macrophages, and suggest that SHIP acts as a gatekeeper for macrophage IL-1β during early innate immune activation.

## Teaser

SHIP negatively regulates synergy for macrophage IL-1β production during NOD2−TLR4 co-stimulation.

## 1. Introduction

Innate immunity forms the first line of defense through physical barriers such as skin and mucosa, and cellular recognition of microbial motifs to initiate inflammatory responses.^1,2^ This recognition is mediated by pattern recognition receptors (PRRs), including Toll-like receptors (TLRs) and NOD-like receptors (NLRs), which sense pathogen-associated molecular patterns (PAMPs), and damage-associated molecular patterns (DAMPs) to initiate downstream signaling.^1,3,4^ Activated innate immune cells coordinate early host defense through the release of inflammatory mediators that drive pathogen clearance^5–8^. Among these cells, macrophages play a central role by integrating signals from multiple PRRs to coordinate cytokine release and antimicrobial activity. In response to TLR or NLR engagement, macrophages activate signaling cascades that promote the production of cytokines such as IL-1β, IL-6, TNFα, and chemokines such as CCL2, CCL3, and CCL4, along with antimicrobial molecules such as nitric oxide and reactive oxygen species^9–12^.

Within the TLR family, TLR4 recognizes lipopolysaccharide (LPS), a glycolipid component of the Gram-negative bacterial outer membrane composed of lipid A, a core oligosaccharide, and an O-antigen polysaccharide.^13,14^ LPS recognition through the co-receptor MD2 facilitates the formation of TLR4–MD2 dimers that initiate downstream signaling.^13,15^ In macrophages and other myeloid cells, the accessory receptor CD14 facilitates LPS-induced internalization of TLR4, enabling signal transduction from both the cell surface and endosomes.^16,17^ Although bacterial LPS is the canonical ligand, TLR4 is also activated by endogenous DAMPs such as HMGB1 and S100A8/A9 during sterile inflammation or tissue damage.^18,19^

NLRs are cytosolic PRRs that are classified into 5 subfamilies, NLRA, NLRB, NLRC, NLRP, and NLRX, based on the structure of their N-terminal domains.^20,21^ Within the NLRC subfamily, NOD2 recognizes muramyl dipeptide (MDP), the minimal bioactive motif of bacterial peptidoglycan present in both Gram-positive and Gram-negative bacteria.^22,23^ NOD2 recognition of MDP is conserved across species, and the receptor is expressed in myeloid cells as well as in epithelial cells, where it contributes to antimicrobial and inflammatory responses^23–25^. Upon ligand recognition, NOD2 undergoes oligomerization and recruits the adaptor kinase RIPK2, leading to downstream activation of the NFκB and MAPK pathways that drive the transcription of proinflammatory cytokines.^26,27^ Co-activation of NOD2 and TLR4 has also been shown to enhance inflammatory responses in primary human monocytes and monocytic cell lines, leading to synergistic increases in IL-1β, IL-6, and TNFα production compared to either ligand alone.^28,29^ IL-1β secretion requires both transcriptional induction of *Il1b* and inflammasome-dependent processing, so enhanced IL-1β production during NOD2 and TLR4 co-stimulation may reflect changes in transcription, inflammasome activation, or both.^30^

Beyond direct antimicrobial activity, macrophages shape immune regulation and tissue homeostasis through cytokine secretion, cell-to-cell crosstalk, and modulation of the inflammatory milieu, and these responses are regulated by pathways that limit excessive inflammation and promote resolution^31–34^. One such negative regulator is the Src homology 2 domain-containing inositol 5′-phosphatase (SHIP), a hematopoietic-specific lipid phosphatase^35–38^. Class I phosphatidylinositol 3-kinases (PI3Ks) phosphorylate phosphatidylinositol (4,5)-bisphosphate (PI(4,5)P_2_) to generate phosphatidylinositol (3,4,5)-trisphosphate (PIP_3_), a second messenger that recruits pleckstrin homology (PH) domain-containing proteins such as AKT to the plasma membrane and activates downstream signaling cascades^39–41^. SHIP blunts this pathway by hydrolyzing PIP_3_ to generate phosphatidylinositol (3,4)-bisphosphate (PI(3,4)P_2_), thereby reducing PIP_3_ availability and negatively regulating PI3K-dependent signaling during immune cell activation.^42,43^ In TLR4 signaling, SHIP upregulation is required for the establishment of endotoxin tolerance in macrophages and *in vivo*.^44^ In addition to its phosphatase activity, SHIP also regulates immune signaling via adaptor functions, noncatalytic mechanisms that involve direct interactions between SHIP and other signaling proteins. Following NOD2 activation, the X-linked inhibitor of apoptosis (XIAP) binds RIPK2 to promote NFκB signaling, and SHIP limits this pathway by disrupting RIPK2–XIAP interactions and reducing NOD2-mediated inflammatory responses^45–47^. We have previously shown that SHIP protein concentrations vary with the macrophage differentiation environment, and are lower in GM-CSF-derived macrophages than in MCSF-derived macrophages.^48^ SHIP protein is also reduced in macrophages treated with IL-4, and its degradation is required for canonical M(IL-4) activation.^48^

Although SHIP has been reported to negatively regulate inflammatory signaling downstream of PRRs, the role of SHIP in macrophage responses to co-stimulation with PRRs remains undefined. Thus, we hypothesized that SHIP limits synergy for cytokine production during co-stimulation with MDP (NOD2) and LPS (TLR4). In bone marrow-derived macrophages (BMDMs), SHIP−/− cells co-stimulated with MDP and LPS had a synergistic increase in IL-1β production, whereas SHIP+/+ cells did not. Reducing SHIP concentrations by culturing BMDMs in different growth factors, by IL-4 treatment, or by SHIP-targeting siRNAs enabled synergistic IL-1β production in response to MDP and LPS. Inhibiting SHIP's catalytic activity did not reproduce this synergy, and synergy was independent of PI3K activity, suggesting that SHIP blocks MDP and LPS synergy for IL-1β through its adaptor function. Consistent with the requirement for NOD2 signaling, pharmacologic NOD2 inhibition suppressed MDP and LPS synergy for IL-1β in SHIP−/− BMDMs. In serial stimulation experiments, IL-1β, IL-6, and TNFα were higher when MDP preceded LPS in both genotypes, but synergy was only observed for IL-1β in SHIP−/− macrophages. These data are consistent with a model in which SHIP's adaptor activity blocks NOD2 signaling and priming of macrophages for increased IL-1β production in response to LPS.

## 2. Materials and methods

### 2.1. Mice

SHIP+/+ and SHIP−/− littermates were generated by breeding heterozygous SHIP (*Inpp5d*^+^/^−^) mice maintained on a mixed C57BL/6 × 129Sv background, and both male and female mice were used at 8 weeks of age for all experiments. Animals were housed in a *Helicobacter*- and specific-pathogen-free animal facility at the BC Children's Hospital Research Institute. All procedures were approved by the University of British Columbia Animal Care Committee (A21-0035 and A21-0218) and conducted in accordance with Canadian Council on Animal Care guidelines.

### 2.2. Macrophage derivation

Bone marrow aspirates were collected from the femora and tibiae of SHIP+/+ and SHIP−/− mice. After a 1 h incubation at 37 °C, nonadherent cells were cultured in IMDM supplemented with 10% FBS, 1% penicillin-streptomycin, 150 μM monothioglycerol, and 5 ng/mL MCSF, GM-CSF, or IL-3 (STEMCELL Technologies, Vancouver, Canada). Cells were maintained at 37 °C in 5% CO_2_ for 10 days with complete media changes on days 4 and 7.

### 2.3. Macrophage stimulation

Bone marrow-derived macrophages (BMDMs) from SHIP+/+ and SHIP−/− mice were either left untreated or stimulated for 24 h with 1 μg/mL MDP (InvivoGen, cat. # tlrl-mdp), 10 ng/mL LPS (*Escherichia coli* serotype 127:B8, Sigma-Aldrich, cat. # L3129), or a combination of MDP and LPS. ATP (5 mM, MP Biomedicals, cat. # 150266) was added during the final hour of stimulation. Supernatants were collected and clarified for cytokine measurements by ELISA. To activate macrophages with IL-4, MCSF-derived BMDMs were cultured in the presence of IL-4 (10 ng/mL, STEMCELL Technologies, Vancouver, Canada) for 72 h prior to stimulation.

For sequential stimulation experiments, MCSF-derived BMDMs were treated with a first ligand (MDP or LPS) for 24 h to prime for the second stimulation, washed 5× with warm PBS, and then cultured for an additional 24 h in fresh media with or without the second ligand (MDP or LPS). Single-ligand (MDP or LPS) and co-stimulation (MDP + LPS) groups were stimulated only during the second 24 h period. ATP was added during the final hour of the second stimulation period.

### 2.4. siRNA knockdown of SHIP

MCSF-derived BMDMs were transfected using Lipofectamine RNAiMAX (Invitrogen, cat. # 13778075) with either a nonsilencing control (nsRNA; Invitrogen, cat. # 4390843) or 1 of 2 SHIP-targeting siRNAs: si1 (Thermo Fisher Scientific, siRNA ID s68357) or si2 (Thermo Fisher Scientific, siRNA ID s68356), as per manufacturer's instructions. Macrophages were cultured for 72 h following transfection before stimulations.

### 2.5. Inhibition experiments

All inhibition experiments were performed in MCSF-derived BMDMs. For SHIP phosphatase inhibition, cells were treated with vehicle control (0.1% ethanol) or 3α-aminocholestane (3AC, MedChemExpress, cat. # HY-19776) at 1, 5, 10, or 20 μM for 30 min prior to stimulation. For PI3K inhibition, cells were treated with vehicle control (0.1% DMSO), LY294002 (10 μM; MedChemExpress, cat. # HY-10108), LY303511 (10 μM; MedChemExpress, cat. # HY-15643A), or wortmannin (100 nM; MedChemExpress, cat. # HY-10197) for 30 min prior to stimulation. For NOD2 inhibition, cells were pretreated with vehicle control (0.1% DMSO), or the selective NOD2 inhibitor GSK717 (10 µM; MedChemExpress, cat. # HY-136555) for 30 min prior to stimulation.

### 2.6. Cytokine measurements

Cytokine concentrations for IL-1β (R&D Systems, cat. # DY401), IL-6 (BD Biosciences, cat. # 555240), and TNFα (BD Biosciences, cat. # 558534) were measured in clarified cell supernatants by ELISA according to the manufacturer's protocols. Samples were assayed in duplicate and values were averaged for each independent experiment.

### 2.7. SDS–PAGE and western blotting

For growth factor (MCSF, GM-CSF, IL-3), IL-4, siRNA, 3AC and PI3K inhibitor experiments, 200,000 cells were loaded per lane for SDS–PAGE. For IL-4 skewing, cells were cultured with IL-4 (10 ng/mL) for 72 h prior to lysis. For siRNA knockdown, cells were transfected with either nonsilencing control or SHIP-targeting siRNAs and cultured for 72 h before lysis. For 3AC and PI3K inhibitor experiments, cells were serum-starved in IMDM containing 0.5% FBS for 4 h. During the final 30 min of starvation, cells were treated with vehicle control (0.1% ethanol) or 3AC (20 µM), or with vehicle control (0.1% DMSO), LY303511 (10 µM), LY294002 (10 µM), or wortmannin (100 nM). Media were then replaced with IMDM containing 10% FBS and MCSF (5 ng/mL), and cells were lysed after 30 min. BMDM lysates were prepared in 2× Laemmli buffer. DNA was sheared by passage through 26-gauge needles, and samples were boiled for 1 min. Proteins were resolved on 8% SDS–polyacrylamide gels and transferred to PVDF membranes using the Invitrogen Power Blotter Semi-Dry Transfer System. Membranes were probed with primary antibodies against SHIP (Santa Cruz Biotechnology, cat. # sc-8425), pAKT (Cell Signaling, cat. # 4060), AKT (Cell Signaling, cat. # 2920), β-actin (Cell Signaling, cat. # 4970) and GAPDH (Fitzgerald Industries International, cat. # 10R-G109A). Secondary antibodies used were IRDye 800CW goat antimouse IgG (LI-COR Biosciences, cat. # 926-32210) and IRDye 800CW goat antirabbit IgG (LI-COR Biosciences, cat. # 926-32211). Blots were imaged on the LI-COR Odyssey Classic system, with either the Precision Plus Protein Kaleidoscope Prestained Protein Standards (BioRad, cat. # 1610375) for Figure 3A, or Chameleon Duo Prestained Protein Ladder (LI-COR Biosciences, cat. # 928-60000) for Figures 4A, 5A, 6A, 7A, as molecular weight markers.

### 2.8. Gene expression

Total RNA was isolated from BMDMs using the RNeasy Plus Mini Kit (QIAGEN, cat. # 74134) according to the manufacturer's instructions. cDNA was generated using iScript Reverse Transcription Supermix (Bio-Rad, cat. # 1708841). Gene expression of *Il1b*, Il6, *Tnf*, *Nod2* and *Tlr4* was quantified by RT-qPCR using SsoAdvanced Universal SYBR Green Supermix (Bio-Rad, cat. # 1725272), with *Gapdh* as the reference gene. SYBR Green primer assays were obtained from Bio-Rad (PrimePCR SYBR Green Assays, cat. # 10025636). The Unique Assay IDs are qMmuCED0045755 for *Il1b*, qMmuCED0045760 for *Il6*, qMmuCED0004141 for *Tnf*, qMmuCID0021902 for *Nod2*, qMmuCID0023548 for *Tlr4*, and qMmuCED0027497 for *Gapdh*.

### 2.9. Caspase-1 activation

Active caspase-1 was measured using the Green Fluorescent FAM-FLICA Caspase-1 (YVAD) Assay Kit (Antibodies Incorporated, cat. # 98) according to the manufacturer's instructions. For microscopy, BMDMs were plated on poly-D-lysine-coated 12 mm round coverslips prior to stimulation as described above. Following stimulation, cells were incubated with FAM-YVAD-FMK to label active caspase-1, counterstained with 4′,6-diamidino-2-phenylindole (DAPI), and imaged at 20× magnification. For fluorescence quantification, BMDMs were plated in black, clear-bottom 96-well plates, stimulated as described above, and incubated with FAM-YVAD-FMK. Fluorescence was measured using a microplate reader, and values are reported as relative fluorescence units (RFU).

### 2.10. LDH cytotoxicity assay

Cytotoxicity was assessed using the LDH-Blue Plus Colorimetric LDH Cytotoxicity Assay Kit (InvivoGen, cat. # rep-ldhv2-1) according to the manufacturer's instructions. Cell culture supernatants were collected after stimulation and incubated with LDH-Blue Plus reagents. Absorbance was measured using a microplate reader, and percent cytotoxicity was calculated relative to maximum LDH release controls.

### 2.11. Statistical analyses

Statistical analyses were performed using one-way ANOVAs with Sidak's corrections for multiple comparisons using GraphPad Prism software, version 8.1. Differences of *P* < 0.05 were considered significant.

## 3. Results

### 3.1. MDP + LPS synergize for IL-1β production in SHIP−/− BMDMs

**Figure 1:**
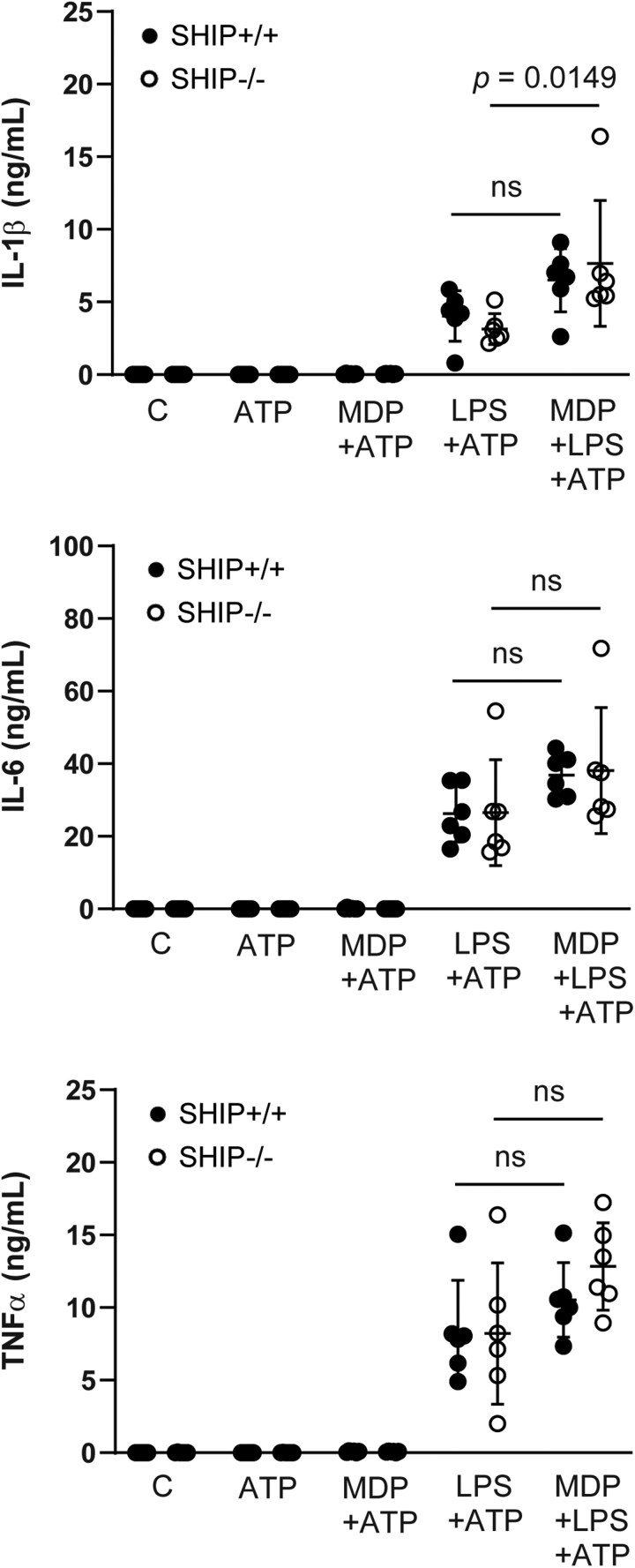
SHIP−/− BMDMs have synergy for IL-1β production in response to co-stimulation with MDP and LPS. MCSF-derived SHIP+/+ and SHIP−/− BMDMs were left untreated (c) or stimulated for 24 h with MDP (1 μg/mL), LPS (10 ng/mL), or MDP + LPS. ATP (5 mM) was added for the final hour. Clarified cell culture supernatants were assayed by ELISA for IL-1β, IL-6, and TNFα. Data are expressed as mean ± SD for *n* = 6. Statistical analyses were performed using a one-way ANOVA with Sidak's multiple comparisons test. *P* values are stated for comparisons indicated. ns = not statistically significant.

**Figure 2:**
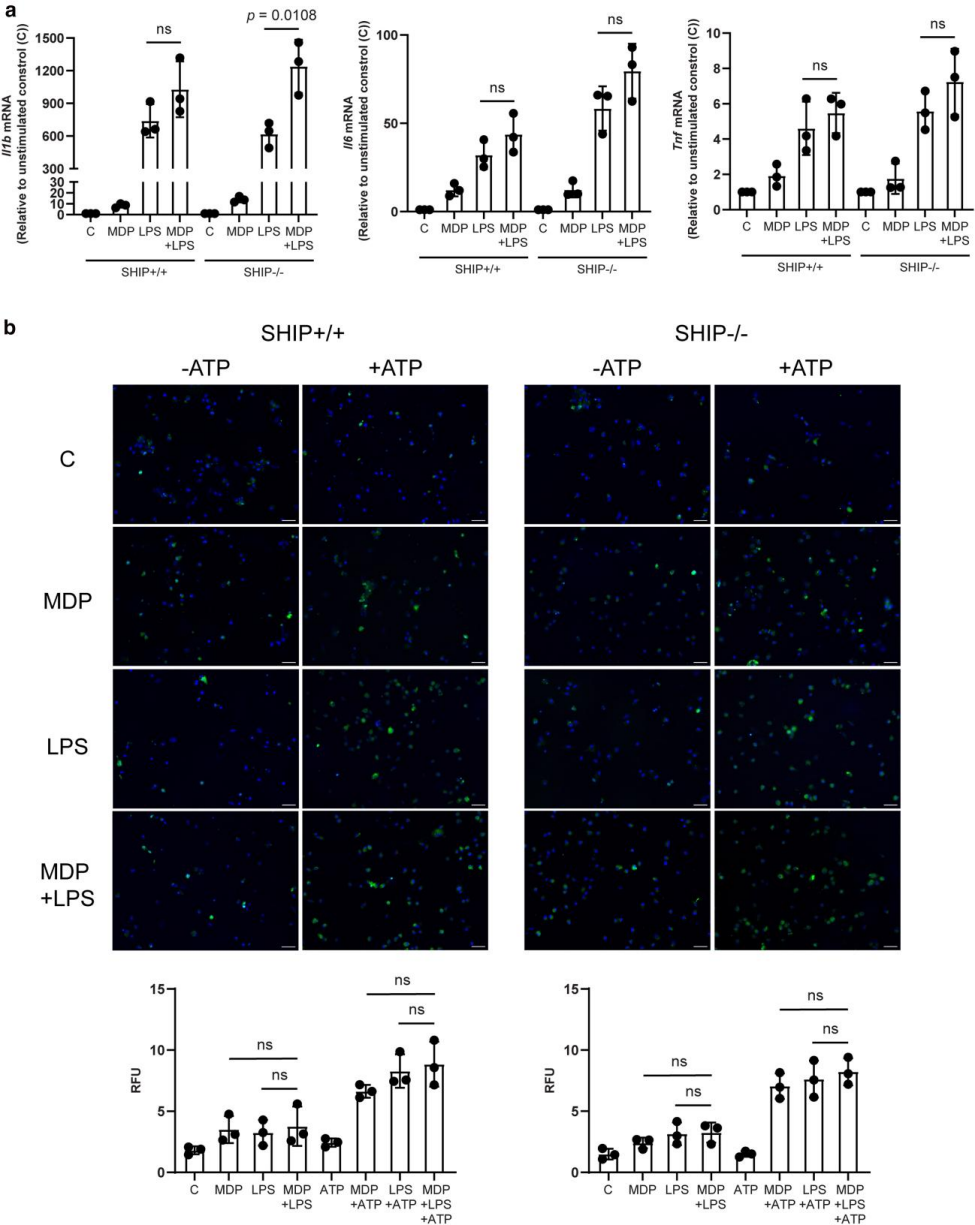
MDP + LPS-induced IL-1β production in SHIP−/− BMDMs is associated with increased *Il1b* expression. MCSF-derived SHIP+/+ and SHIP−/− BMDMs were stimulated with MDP (1 μg/mL), LPS (10 ng/mL), or MDP + LPS for 24 h. a) *Il1b*, *Il6*, and *Tnf* expression were measured by RT-qPCR, normalized to *Gapdh*, and reported relative to unstimulated control. b) Active caspase-1 was measured using FAM-FLICA staining in the absence or presence of ATP. Active caspase-1 staining was visualized by fluorescence microscopy, and fluorescence was quantified as relative fluorescence units (RFU). Scale bars = 50 μm. Data are expressed as mean ± SD for *n* = 3. Statistical analyses were performed using a one-way ANOVA with Sidak's multiple comparisons test. *P* values are stated for comparisons indicated. ns = not statistically significant.

SHIP regulates innate immune signaling downstream of both NOD2 and TLR4, and loss of SHIP in macrophages has been associated with increased cytokine responses.^44,45,49^ To assess how SHIP deficiency affects responses to combined NOD2 and TLR4 stimulation, MCSF-derived BMDMs from SHIP+/+ and SHIP−/− mice were stimulated with MDP, LPS, or both for 24 h. ATP was added during the final hour of stimulation to activate the inflammasome, which is required for IL-1β processing and secretion (Fig. 1). Consistent with a synergistic response, MDP + LPS stimulation induced significantly more IL-1β than LPS alone in SHIP−/− BMDMs, whereas no such effect was observed in SHIP+/+ BMDMs. IL-6 and TNFα were not significantly increased by co-stimulation in either genotype. These findings suggest that SHIP limits macrophage cytokine responses to NOD2 and TLR4 co-stimulation, with SHIP deficiency being required for synergy for IL-1β production. To determine whether the increased IL-1β observed in SHIP−/− BMDMs was associated with increased mRNA expression, SHIP+/+ and SHIP−/− BMDMs were stimulated with MDP, LPS, or MDP + LPS, and *Il1b*, *Il6*, and *Tnf* expression were measured. MDP + LPS stimulation significantly increased *Il1b* expression compared to LPS alone in SHIP−/− BMDMs, whereas this difference was not significant in SHIP+/+ BMDMs (Fig. 2a). In contrast, *Il6* and *Tnf* expression were not significantly increased by MDP + LPS compared to LPS in either genotype. Inflammasome activation was then assessed by measuring active caspase-1 in BMDMs stimulated with MDP, LPS, or MDP + LPS in the absence or presence of ATP (Fig. 2b). Active caspase-1 staining was visualized by microscopy, and fluorescence was quantified. MDP + LPS did not significantly increase active caspase-1 staining or fluorescence compared to MDP or LPS alone in either genotype, regardless of ATP treatment. Cytotoxicity was assessed by LDH release and was not significantly increased by MDP + LPS compared to LPS alone in either genotype (Figure S1). Together, these findings suggest that MDP + LPS-induced synergy for IL-1β production in SHIP−/− BMDMs is associated with increased *Il1b* expression.

### 3.2. SHIP protein concentrations are inversely related to (MDP + LPS)-induced synergy for IL-1β production in SHIP+/+ BMDMs

**Figure 3:**
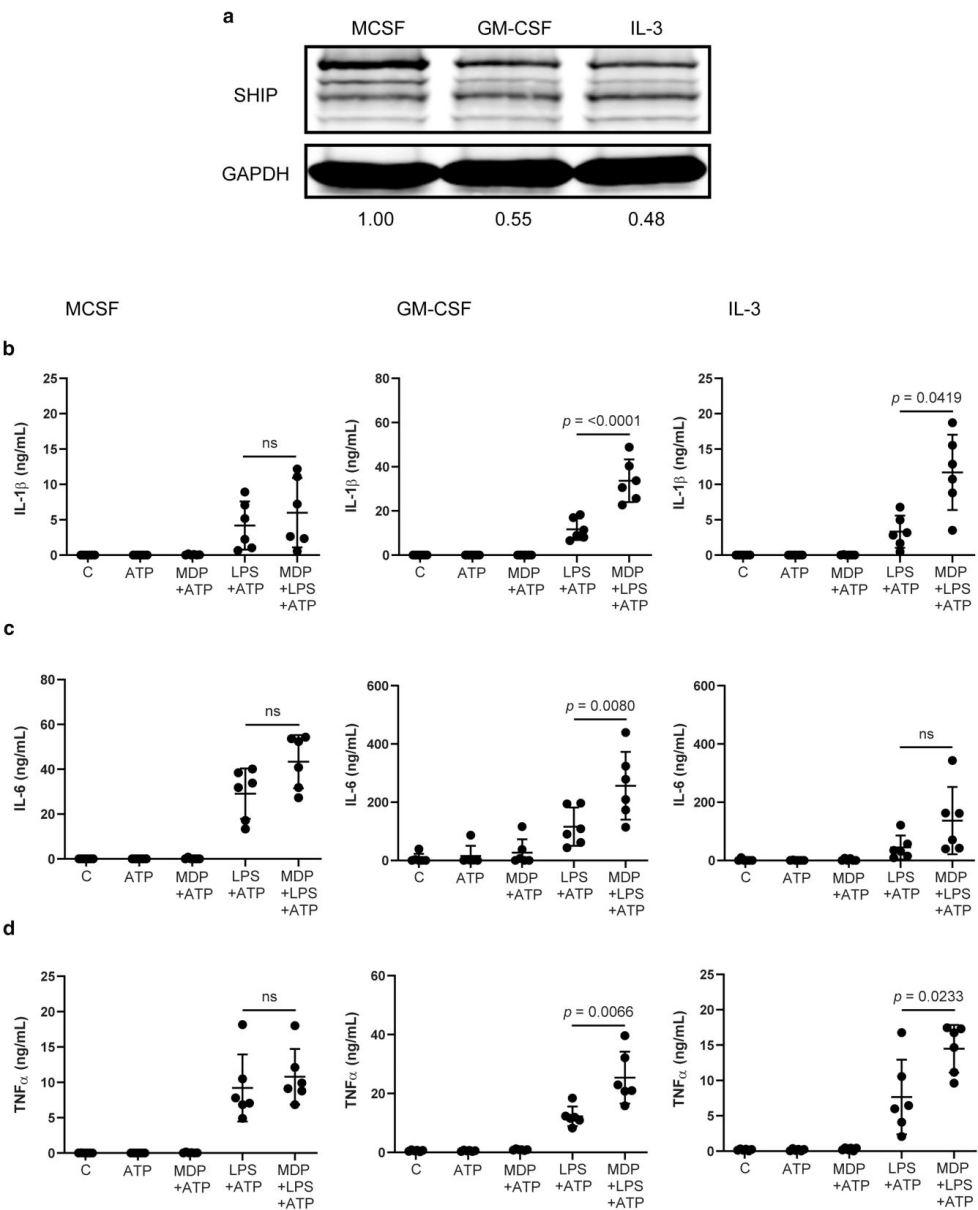
SHIP protein concentrations in SHIP+/+ BMDMs derived with different macrophage growth factors inversely correlate with (MDP + LPS)-induced synergy for IL-1β production. a) Western blot of SHIP protein in SHIP+/+ BMDMs differentiated using MCSF, GM-CSF, or IL-3. GAPDH is a loading control. (b–d) SHIP+/+ BMDMs were left untreated (control, (c) or stimulated with MDP (1 μg/mL), LPS (10 ng/mL), or MDP + LPS for 24 h. ATP (5 mM) was added for the final hour. Culture supernatants were collected, clarified, and assayed by ELISA for (b) IL-1β, (c) IL-6, and (d) TNFα. Data are expressed as mean ± SD for *n* = 3 for (a), and *n* = 6 for (b–d). Statistical analyses were performed using a one-way ANOVA with Sidak's multiple comparisons test. *P* values are stated for comparisons indicated. ns = not statistically significant.

SHIP is a lipid phosphatase that regulates PI3K signaling, and differences in macrophage differentiation can affect SHIP protein abundance.^38,48^ GM-CSF-derived BMDMs have previously been reported to have less SHIP compared to MCSF-derived BMDMs.^48^ To investigate the impact of SHIP concentrations on (MDP + LPS)-induced synergy, SHIP+/+ macrophages were derived from bone marrow aspirates with 3 different macrophage growth factors, MCSF, GM-CSF, or IL-3. Whole cell lysates from SHIP+/+ BMDMs derived in each condition were probed for SHIP by western blot, using GAPDH as a loading control. GM-CSF- and IL-3-derived BMDMs had 0.55 and 0.48-fold SHIP protein compared to MCSF-derived BMDMs, respectively (Fig. 3a). Following confirmation of reduced SHIP protein concentrations in GM-CSF- and IL-3-derived BMDMs, cytokine production in response to NOD2 and TLR4 stimulation was measured. BMDMs were stimulated with MDP, LPS, or MDP + LPS for 24 h with the addition of ATP treatment for the final hour, and IL-1β was measured in the supernatants by ELISA (Fig. 3b). MCSF-derived BMDMs did not show synergy for IL-1β production when co-stimulated with MDP + LPS. In contrast, both GM-CSF- and IL-3-derived BMDMs, which have lower SHIP concentrations, produced more IL-1β in response to MDP + LPS compared to LPS alone, suggesting that high SHIP concentrations in MCSF-derived macrophages block synergy. IL-6 was also measured following MDP, LPS, or MDP + LPS stimulation (Fig. 3c). Synergy was limited to GM-CSF-derived cells, with no significant differences detected in MCSF- or IL-3-derived cells. TNFα production in response to MDP + LPS stimulation followed a similar pattern to IL-1β, with synergy observed in GM-CSF- and IL-3-derived BMDMs, but not in MCSF-derived BMDMs (Fig. 3d).

Cytokine mRNA expression was next assessed in MCSF-, GM-CSF-, and IL-3-derived BMDMs following stimulation with MDP, LPS, or MDP + LPS. MCSF-derived BMDMs did not show significant differences in *Il1b*, *Il6*, or *Tnf* expression between LPS and MDP + LPS stimulation (Figure S2a). In contrast, MDP + LPS stimulation increased *Il1b*, *Il6*, and *Tnf* compared to LPS in GM-CSF-derived BMDMs. In IL-3-derived BMDMs, MDP + LPS increased *Il1b* expression compared to LPS alone, whereas *Il6* and *Tnf* expression were not significantly different. Since receptor expression could influence responsiveness to MDP and LPS, *Nod2* and *Tlr4* expression were also measured under the same stimulation conditions (Figure S2b). LPS alone was sufficient to increase *Nod2* expression. *Nod2* and *Tlr4* were not increased by MDP + LPS co-stimulation compared to LPS alone in any of the BMDM populations. Active caspase-1 was then measured in BMDMs stimulated with MDP, LPS, or MDP + LPS. Across the BMDM populations examined, MDP + LPS did not increase caspase-1 activation compared to LPS (Figure S2c). To assess whether growth factor-dependent differences in cytokine responsiveness were maintained in the absence of SHIP, MCSF-, GM-CSF-, and IL-3-derived SHIP−/− BMDMs were stimulated with MDP, LPS, or MDP + LPS. MDP + LPS increased IL-1β production compared to LPS alone in SHIP−/− BMDMs derived with each growth factor, including MCSF (Figure S3). As in SHIP+/+ BMDMS, IL-6 was increased by MDP + LPS in GM-CSF, and also increased in IL-3-derived SHIP−/− BMDMs, but not in MCSF-derived SHIP−/− BMDMs. In the absence of SHIP, TNFα was not significantly increased by MDP + LPS in any of the BMDM populations. Together, these findings support an inverse relationship between SHIP protein concentration and the capacity of BMDMs to mount IL-1β responses to MDP + LPS co-stimulation, but also indicate that growth factor-dependent differentiation can shape IL-6 and TNFα responses independently of SHIP.

### 3.3. IL-4 activation reduces SHIP protein concentrations in MCSF-derived BMDMs and leads to (MDP + LPS)-induced synergy for proinflammatory cytokine production

**Figure 4:**
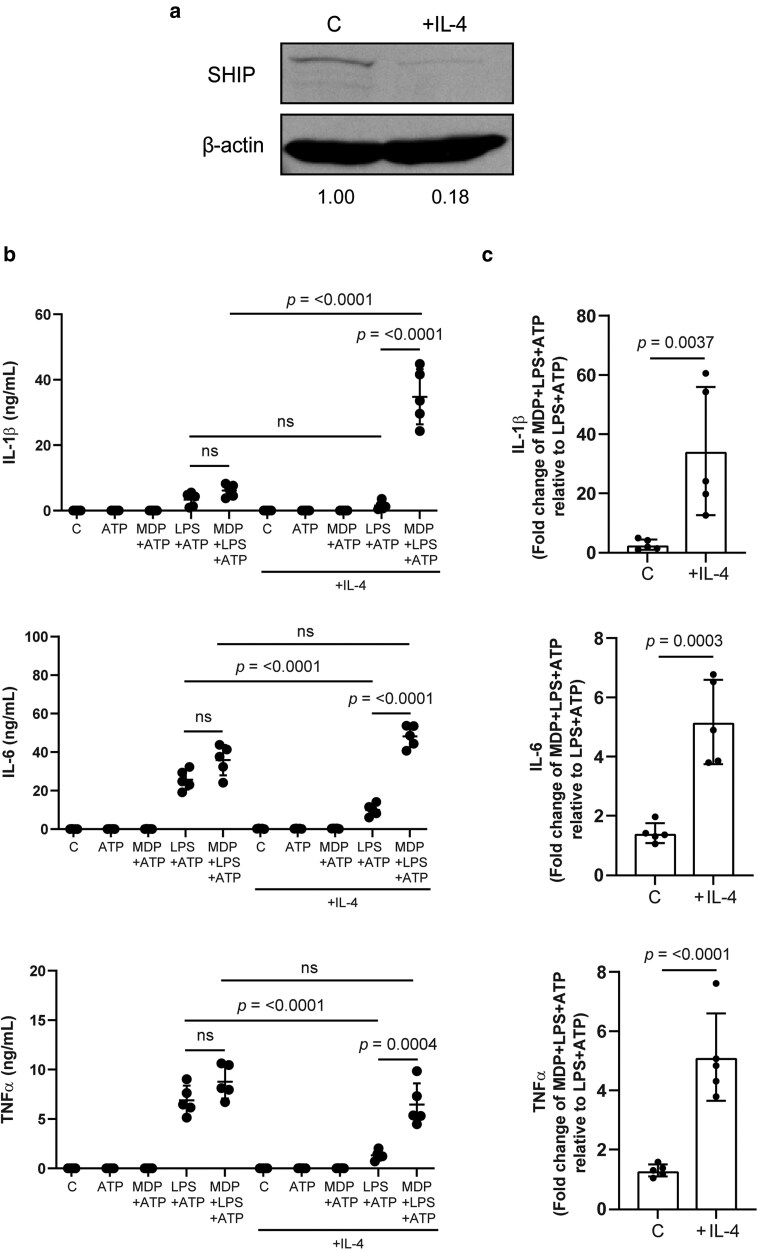
IL-4 treatment reduces SHIP protein concentrations and increases (MDP + LPS)-induced synergy for proinflammatory cytokine production in SHIP+/+ MCSF-derived BMDMs. a) Western blot of SHIP protein in SHIP+/+ MCSF-derived BMDMs following 72 h treatment with or without IL-4 (10 ng/mL). β-actin is a loading control. b) SHIP+/+ BMDMs were pretreated ± IL-4 for 72 h and then were untreated (control, (c) or stimulated with MDP (1 μg/mL), LPS (10 ng/mL), or MDP + LPS for 24 h. ATP (5 mM) was added for the final hour. Clarified cell culture supernatants were assayed by ELISA for IL-1β, IL-6, and TNFα. c) Fold change in cytokine production between MDP + LPS and LPS treatment groups. Data are expressed as mean ± SD for *n* = 3 for (a), and *n* = 5 for (b) and (c). Statistical analyses were performed using a one-way ANOVA with Sidak's multiple comparisons test. *P* values are stated for comparisons indicated. ns = not statistically significant.

IL-4 stimulation leads to SHIP degradation in macrophages and drives M(IL-4) activation, which is typically associated with lower production of proinflammatory cytokines in response to innate immune stimuli.^48,50^ Thus, we asked whether M(IL-4) macrophages were capable of mounting a synergistic proinflammatory cytokine response to MDP and LPS. SHIP+/+ MCSF-derived BMDMs were cultured with IL-4 for 72 h and compared to the untreated control. Western blot analysis showed that SHIP protein was reduced by IL-4 treatment (Fig. 4a). Densitometric analysis, normalized to the untreated control, showed relative SHIP protein of 1.00 in untreated cells and 0.18 in IL-4-treated cells. Following confirmation of lower SHIP concentrations in M(IL-4) BMDMs, cytokine responses were assessed in macrophages stimulated with MDP, LPS, or MDP + LPS for 24 h, with ATP added during the final hour (Fig. 4b). MCSF-derived BMDMs and M(IL-4) produced little or no IL-1β, IL-6, or TNFα in response to MDP stimulation. Importantly, and as previously reported, M(IL-4) produced lower amounts of proinflammatory cytokines in response to LPS than BMDMs that were not treated with IL-4 (Fig. 4b). In untreated MCSF-derived BMDMs, MDP + LPS co-stimulation did not significantly increase IL-1β, IL-6, or TNFα production compared to LPS alone. Contrary to expectations for reduced cytokine responses by M(IL-4), IL-4-treated BMDMs had significantly increased production of all 3 cytokines following MDP + LPS co-stimulation, indicating that prior exposure to IL-4 is sufficient to enable synergy for IL-1β, IL-6, and TNFα production by macrophages and dramatically increased the magnitude of IL-1β production in response to co-stimulation with MDP + LPS. The fold increase in cytokine production comparing MDP + LPS stimulation relative to LPS alone was calculated for each biological replicate and compared between untreated and IL-4-treated macrophages (Fig. 4c). IL-4 treatment significantly increased the fold change for all 3 cytokines, consistent with increased responsiveness of IL-4-treated BMDMs to MDP + LPS co-stimulation. The effect of IL-4 treatment on cytokine mRNA expression was then examined. Untreated MCSF-derived BMDMs did not show significant differences in *Il1b*, *Il6*, or *Tnf* expression between LPS and MDP + LPS stimulation (Figure S4a). In contrast, MDP + LPS stimulation increased *Il1b*, *Il6*, and *Tnf* compared to LPS alone in IL-4-treated BMDMs. Active caspase-1 was measured in untreated and IL-4-treated cells, and MDP + LPS did not increase caspase-1 activation compared to LPS (Figure S4b). To assess whether IL-4-dependent enhancement of cytokine responses required SHIP, the same stimulation conditions were tested in SHIP−/− BMDMs. Untreated SHIP−/− BMDMs demonstrated synergy for IL-1β production in the absence of IL-4 treatment, which was further enhanced by treatment with IL-4 (Figure S5). Moreover, the magnitude of IL-6 and TNFα production in M(IL-4) BMDMs was dramatically increased in response to co-stimulation (Figure S5). Together, these data show that IL-4 treatment enables strong MDP + LPS-induced production of IL-1β, IL-6, and TNFα in MCSF-derived BMDMs regardless of SHIP genotype, indicating that the IL-4-induced phenotype cannot be explained solely by SHIP loss. These findings suggest that IL-4 promotes a macrophage activation state that supports enhanced cytokine production following MDP + LPS co-stimulation, with SHIP reduction potentially contributing to this response in SHIP+/+ cells.

### 3.4. SHIP knockdown selectively enhances IL-1β responses to MDP + LPS co-stimulation

**Figure 5:**
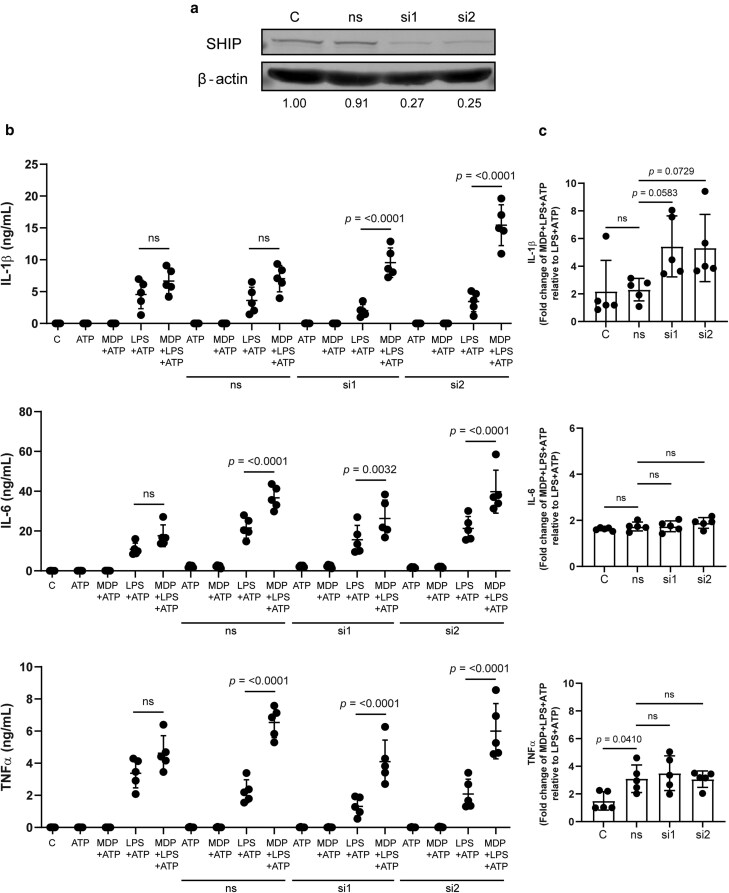
SHIP siRNA knockdown enables (MDP + LPS)-induced synergy for IL-1β production in SHIP+/+ MCSF-derived BMDMs. a) Western blot of SHIP protein in MCSF-derived SHIP+/+ BMDMs treated with nonsilencing RNA control (ns) or 2 independent SHIP-targeting siRNAs (si1 and si2) for 72 h. β-actin is a loading control. b) SHIP+/+ BMDMs were left untreated (control, (c) or stimulated with MDP (1 μg/mL), LPS (10 ng/mL), or MDP + LPS for 24 h following transfection. ATP (5 mM) was added for the final hour. Clarified cell culture supernatants were collected and assayed by ELISA for IL-1β, IL-6, and TNFα. c) Fold change of MDP + LPS to LPS for cytokine production. Data are expressed as mean ± SD for *n* = 3 for (a), and n = 5 for (b) and (c). Statistical analyses were performed using a one-way ANOVA with Sidak's multiple comparisons test. *P* values are stated for comparisons indicated. ns = not statistically significant.

Thus far, our data have shown that low SHIP protein concentrations correlate with MDP and LPS synergy for IL-1β production. To directly assess SHIP's role in this response, we used siRNA to specifically reduce SHIP protein concentrations in macrophages. SHIP+/+ MCSF-derived BMDMs were transfected with either a nonsilencing control RNA (ns), or 1 of 2 independent SHIP-targeting siRNAs (si1 or si2), and cultured for 72 h. Both SHIP-targeting siRNAs effectively reduced SHIP protein compared to untreated and ns controls (Fig. 5a). Densitometric analysis, normalized to the untreated control, showed relative SHIP protein of 0.91 in ns-transfected cells, 0.27 in si1-transfected cells, and 0.25 in si2-transfected cells. Untransfected BMDMs and macrophages transfected with either ns, si1, or si2 were stimulated with MDP, LPS, or MDP + LPS, and their cytokine production was measured by ELISA (Fig. 5b). For IL-1β, MDP + LPS significantly increased cytokine production compared to LPS alone in both si1− and si2-transfected cells, but not in untransfected or ns controls. For IL-6 and TNFα, significant increases between MDP + LPS stimulation versus LPS stimulation were observed in ns-, si1-, and si2-transfected cells, but not in untransfected BMDMs. These results suggest that the transfection process itself had increased responsiveness to MDP + LPS for IL-6 and TNFα, as synergy was observed even in cells transfected with the ns control. In contrast, IL-1β required SHIP knockdown to elicit a synergistic response, which is consistent with a model in which SHIP limits IL-1β production downstream of NOD2 and TLR4 co-stimulation. To assess whether SHIP knockdown affected the magnitude of the cytokine response to co-stimulation, the fold change comparing cytokine production by MDP + LPS relative to LPS was calculated for each cytokine (Fig. 5c). Fold change in IL-1β was higher in both si1− and si2-transfected cells compared to the ns control, although these differences did not reach statistical significance. Fold changes in IL-6 were consistent across all groups, with no significant differences observed. For TNFα, fold change was significantly higher in the ns control compared to untreated cells, but knockdown with si1 or si2 did not increase this response. *Il1b*, *Il6*, and *Tnf* expression were then assessed in untransfected, ns-, si1-, and si2-transfected BMDMs. MDP + LPS stimulation significantly increased *Il1b* expression compared to LPS in si1− and si2-transfected BMDMs, but not in untransfected or ns-transfected controls (Figure S6a). In contrast, *Il6* and *Tnf* expression were not significantly different between LPS and MDP + LPS stimulation in any of the transfection groups. Across these conditions, MDP + LPS did not increase caspase-1 activation compared to LPS alone (Figure S6b). Together, these findings suggest that SHIP knockdown selectively enhances (MDP + LPS)-induced synergy for IL-1β transcription.

### 3.5. Inhibition of SHIP phosphatase activity does not promote (MDP + LPS)-induced cytokine synergy in MCSF-derived macrophages

**Figure 6:**
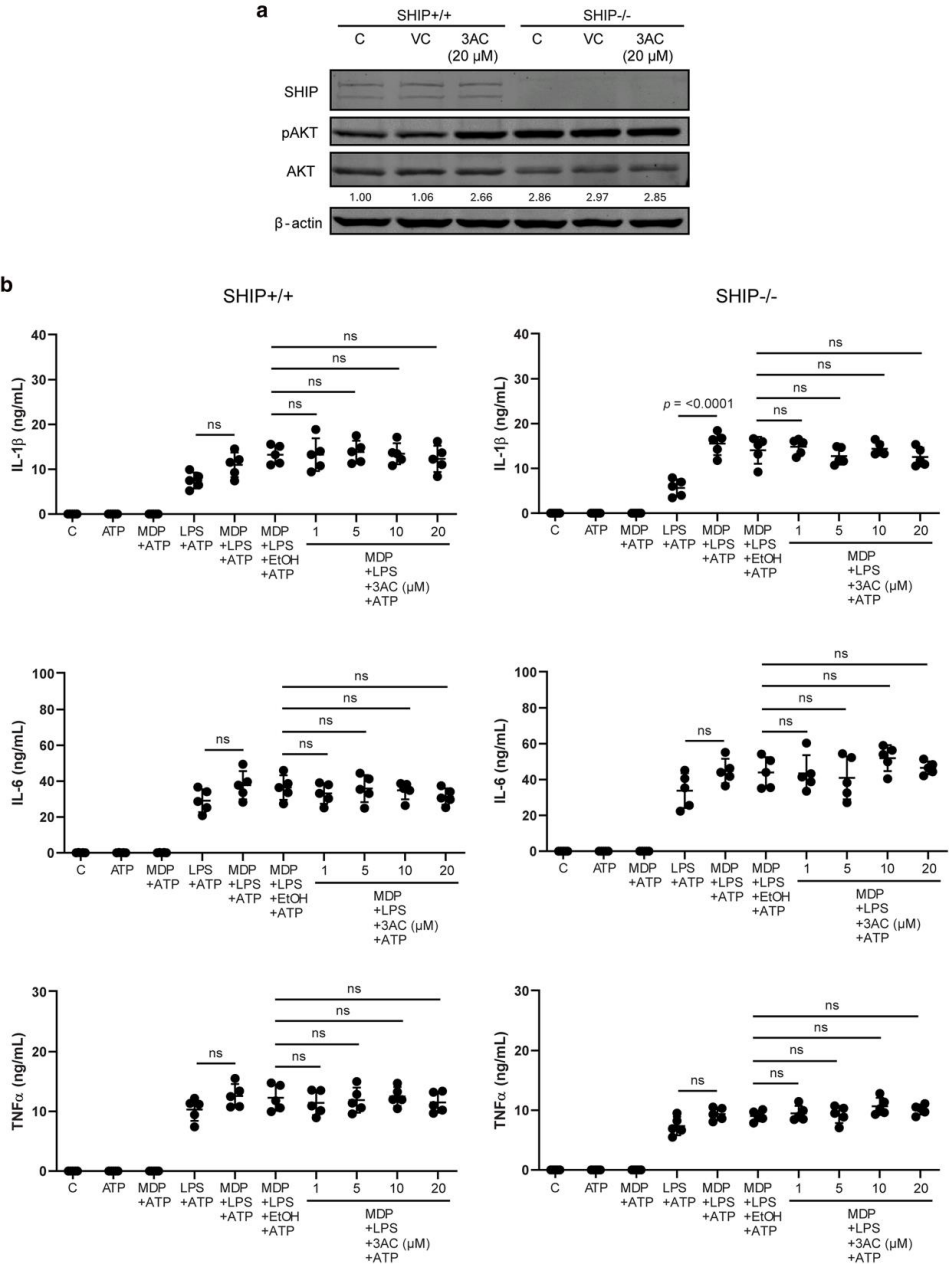
Inhibition of SHIP activity does not increase (MDP + LPS)-induced cytokine production. a) Western blot of phosphorylated AKT (pAKT) and total AKT in SHIP+/+ and SHIP−/− MCSF-derived BMDMs treated with vehicle control (VC, 0.1% ethanol) or the SHIP inhibitor 3AC (20 μM) for 30 min. β-actin is a loading control. b) SHIP+/+ and SHIP−/− BMDMs were pretreated with vehicle control or increasing concentrations of 3AC (1, 5, 10, or 20 μM) for 30 min prior to stimulation with MDP (1 μg/mL), LPS (10 ng/mL), or MDP + LPS for 24 h. ATP (5 mM) was added for the final hour. Clarified cell culture supernatants were collected and assayed by ELISA for IL-1β, IL-6, and TNFα. Data are expressed as mean ± SD for *n* = 3 for (a), and *n* = 5 for (b). Statistical analyses were performed using a one-way ANOVA with Sidak's multiple comparisons test. *P* values are stated for comparisons indicated. ns = not statistically significant.

Next, we asked whether SHIP's phosphatase activity is required to block (MDP + LPS)-induced synergy for IL-1β production. SHIP's catalytic activity was inhibited using 3α-aminocholestane (3AC), a small-molecule inhibitor that blocks the conversion of PI(3,4,5)P_3_ to PI(3,4)P_2_ by SHIP, therefore increasing PI(3,4,5)P_3_ concentrations and AKT activation.^51^ To confirm 3AC activity, MCSF-derived BMDMs were serum-starved for 4 h, treated with vehicle control (0.1% ethanol) or 3AC (20 μM) during the final 30 min, and then cultured in complete medium containing MCSF for 30 min before lysis. Phosphorylated AKT (pAKT), a downstream target of PI3K signaling negatively regulated by SHIP, was assessed by Western blot (Fig. 6a).^52^ In SHIP+/+ BMDMs, there was more pAKT following 3AC treatment compared to vehicle control, whereas SHIP−/− BMDMs had higher basal pAKT that was not further increased by 3AC. Quantification by densitometry, normalized to untreated SHIP+/+ showed relative pAKT intensities of 1.00 (control, C), 1.06 (vehicle control, VC), and 2.66 (3AC) in SHIP+/+ BMDMs, and 2.86 (C), 2.97 (VC), and 2.85 (3AC) in SHIP−/− BMDMs. These results confirm that 3AC is functionally active at 20 μM and increases phosphorylation of AKT. SHIP+/+ and SHIP−/− BMDMs were stimulated with MDP, LPS, or MDP + LPS for 24 h with ATP addition during the final hour in the presence of vehicle control (0.1% ethanol), or increasing concentrations of 3AC (1, 5, 10, or 20 μM). Cytokine concentrations were measured in macrophage supernatants by ELISA (Fig. 6b). In SHIP+/+ BMDMs, IL-1β, IL-6, and TNFα production in response to MDP + LPS was not significantly increased by any of the tested concentrations of 3AC compared to the vehicle control. As expected, cytokine production in SHIP−/− BMDMs was unaffected by 3AC treatment since these cells lack SHIP. 3AC did not significantly alter *Il1b*, *Il6*, or *Tnf* mRNA expression (Figure S7a), caspase-1 activation (Figure S7B) or cytotoxicity (Figure S8) compared to vehicle control. Inhibition of SHIP's phosphatase activity did not recapitulate the effects of SHIP knockdown, suggesting that (MDP + LPS)-induced synergy for IL-1β production is independent of SHIP activity.

### 3.6. PI3K activity is dispensable for regulating (MDP + LPS)-induced cytokine responses in SHIP−/− BMDMs

**Figure 7:**
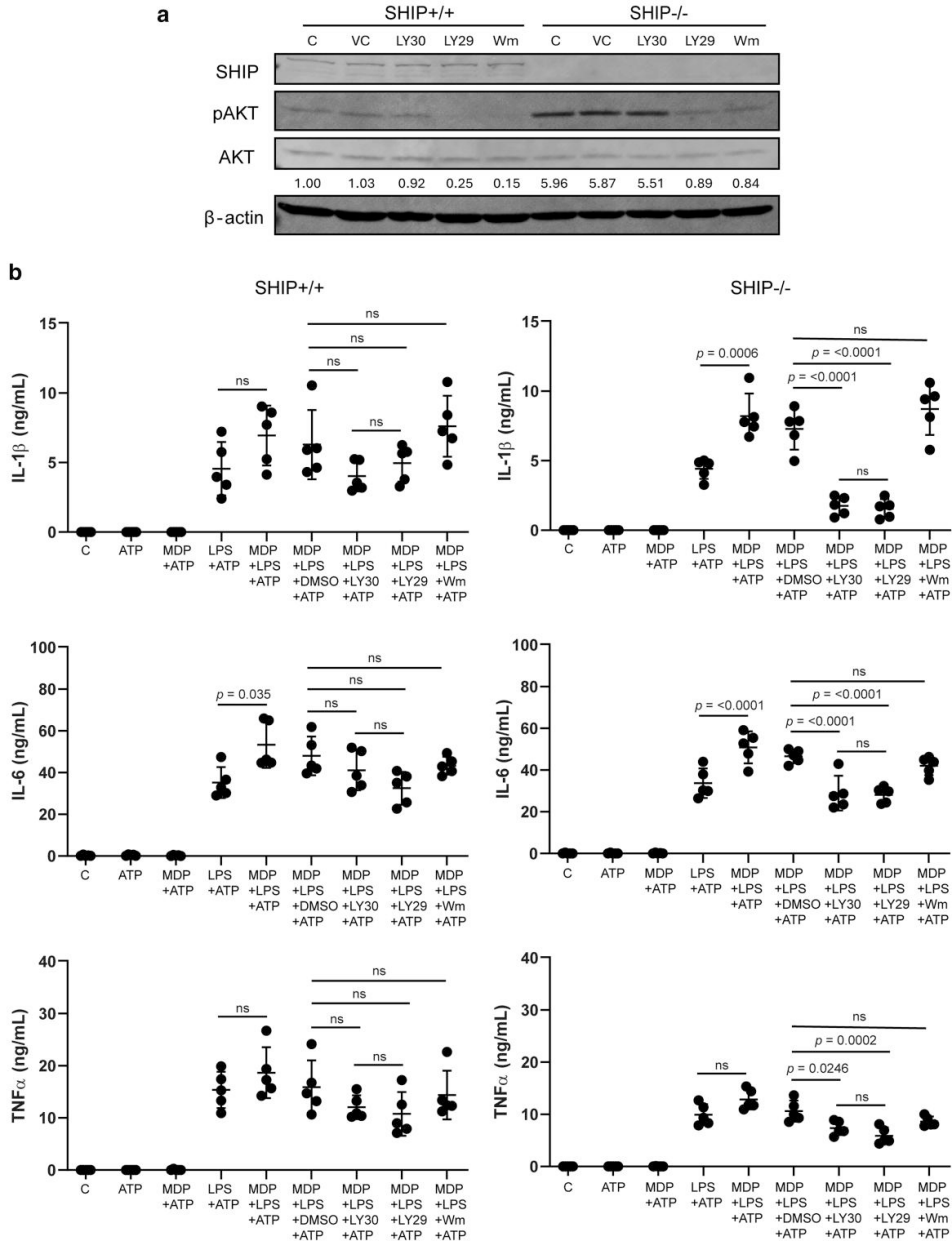
Inhibition of PI3K activity does not reduce (MDP + LPS)-induced synergy for IL-1β production in SHIP−/− BMDMs. a) Western blot of phosphorylated AKT (pAKT) and total AKT in SHIP+/+ and SHIP−/− MCSF-derived BMDMs following treatment with vehicle control (VC, 0.1% DMSO), LY303511 (LY30, negative control for PI3K inhibition, 10 μM), LY294002 (LY29, 10 μM), or wortmannin (Wm, 100 nM) for 30 min. β-actin is a loading control. b) SHIP+/+ and SHIP−/− BMDMs were pretreated with vehicle control (0.1% DMSO), LY30 (10 μM), LY29 (10 μM), or Wm (100 nM) for 30 min prior to stimulation with MDP (1 μg/mL), LPS (10 ng/mL), or MDP + LPS for 24 h. ATP (5 mM) was added for the final hour. Clarified cell culture supernatants were collected and assayed by ELISA for IL-1β, IL-6, and TNFα. Data are expressed as mean ± SD for *n* = 3 for (a) and *n* = 5 for (b). Statistical analyses were performed using a one-way ANOVA with Sidak's multiple comparisons test. *P* values are stated for comparisons indicated. ns = not statistically significant.

Since SHIP's catalytic activity blocks PI3K signaling by hydrolyzing PI(3,4,5)P_3_, we next tested whether PI3K activity was required for (MDP + LPS)-induced IL-1β synergy to confirm that SHIP's regulatory role was independent of its phosphatase function.^53,54^ MCSF-derived SHIP+/+ and SHIP−/− BMDMs were treated with 2 pan-PI3K inhibitors, LY294002 (LY29) and wortmannin (Wm), as well as LY303511 (LY30), a structural analog of LY29 that does not inhibit PI3K activity.^55^ To confirm inhibition, MCSF-derived BMDMs were serum-starved for 4 h and treated during the final 30 min with LY30 (10 µM), LY29 (10 µM), or Wm (100 nM). Macrophages were then incubated for 30 min in complete medium containing MCSF before lysis. pAKT was measured by Western blot as a downstream marker of PI3K signaling (Fig. 7a). In SHIP+/+ BMDMs, pAKT was reduced by treatment with LY29 or Wm, but not in response to vehicle control (VC) or LY30. SHIP−/− BMDMs had higher baseline pAKT compared to untreated SHIP+/+ cells, consistent with loss of SHIP activity, and similarly responded to LY29 and Wm with reduced pAKT, whereas VC or LY30 had no effect. Densitometric analysis, normalized to untreated SHIP+/+ (C), showed relative pAKT intensities of 1.00 (C), 1.03 (VC), 0.92 (LY30), 0.25 (LY29), and 0.15 (Wm) in SHIP+/+ BMDMs, and 5.96 (C), 5.87 (VC), 5.51 (LY30), 0.89 (LY29), and 0.84 (Wm) in SHIP−/− BMDMs. These results confirm that LY29 and Wm effectively reduce AKT phosphorylation in macrophages, consistent with inhibition of PI3K signaling. Cytokine production in response to MDP + LPS stimulation was measured in the presence of PI3K inhibitors. SHIP+/+ and SHIP−/− BMDMs were stimulated with MDP, LPS, or MDP + LPS for 24 h, with ATP addition during the final hour, in the presence of VC (0.1% DMSO), LY30 (10 µM), LY29 (10 µM), or Wm (100 nM), and supernatants were analyzed for IL-1β, IL-6, and TNFα by ELISA (Fig. 7b). In SHIP+/+ BMDMs, PI3K inhibitors had no significant effect on IL-1β, IL-6, or TNFα production following MDP + LPS stimulation. In SHIP−/− BMDMs, IL-1β, IL-6, and TNFα concentrations were reduced by both LY29 and LY30, whereas Wm had no effect. Since LY30 does not inhibit PI3K activity, the reduction in cytokine production observed with LY29 cannot be attributed to PI3K blockade, and this result was further supported by the lack of effect of Wm. LY30, LY29, and Wm did not significantly affect *Il1b*, *Il6*, or *Tnf* mRNA expression (Figure S7a), caspase-1 activation (Figure S7b), or cytotoxicity (Figure S8). Together, these data suggest that the reduction in cytokine production by LY29 is not due to inhibition of PI3K, and may instead reflect off-target activity shared with LY30. Overall, these results support a model in which SHIP blocks MDP + LPS-induced IL-1β production independently of its phosphatase activity.

### 3.7. NOD2 inhibition reduces IL-1β synergy in SHIP−/− BMDMs

**Figure 8:**
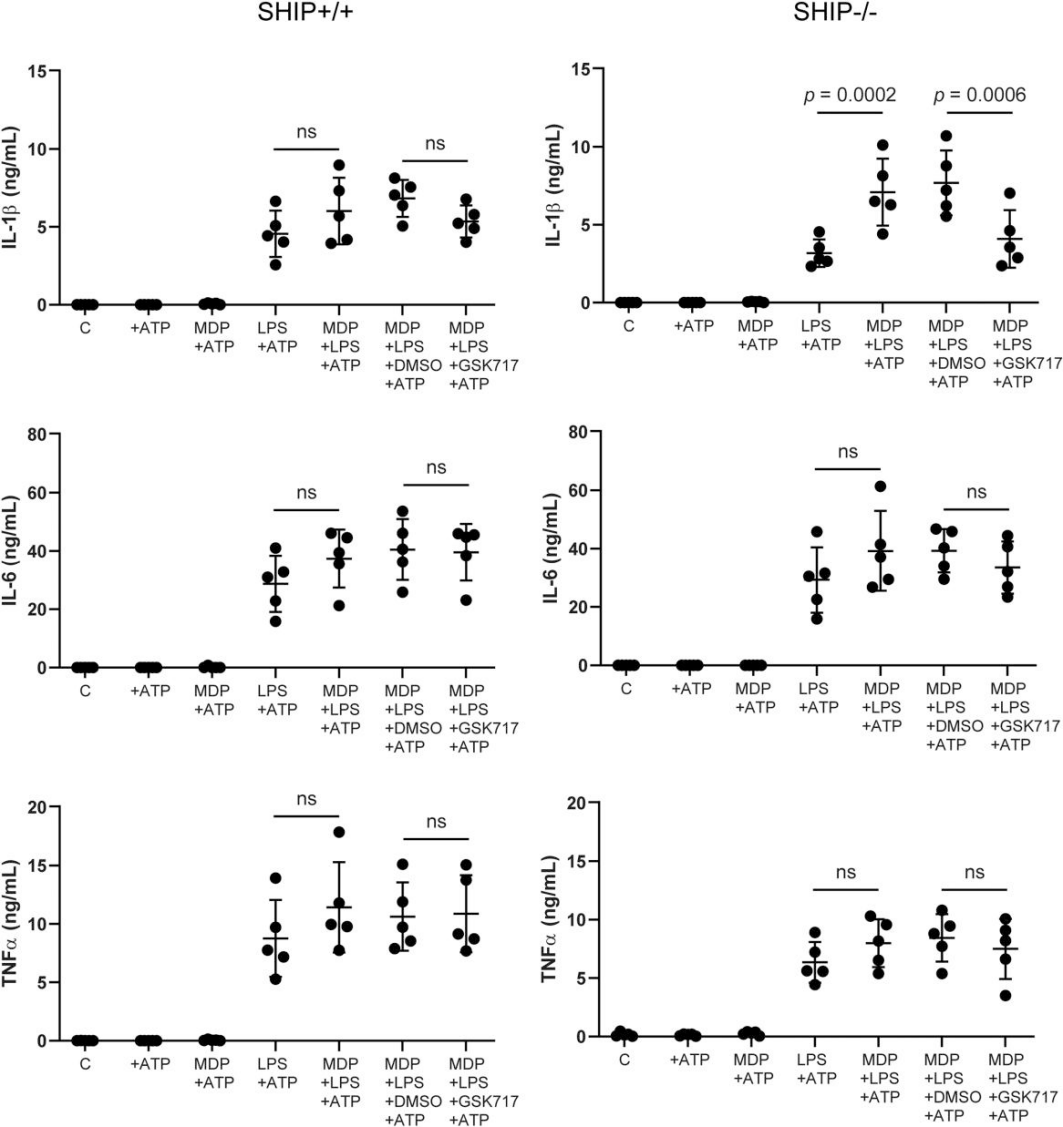
NOD2 inhibition reduces (MDP + LPS)-induced IL-1β production in SHIP−/− BMDMs. SHIP+/+ and SHIP−/− MCSF-derived BMDMs were pretreated with vehicle control (0.1% DMSO) or the NOD2 inhibitor GSK717 (10 µM) for 30 min prior to stimulation with MDP (1 µg/mL), LPS (10 ng/mL), or MDP + LPS for 24 h. ATP (5 mM) was added during the final hour. Clarified cell supernatants were assayed by ELISA for IL-1β, IL-6, and TNFα. Data are expressed as mean ± SD of *n* = 5. Statistical analyses were performed using a one-way ANOVA with Sidak's multiple comparisons test. *P* values are stated for comparisons indicated. ns = not statistically significant.

SHIP has been reported to regulate NOD2 signaling through its role as an adaptor, ie protein-protein interactions between SHIP and XIAP, which sequesters XIAP from its function downstream of NOD2 ligation.^45^ Thus, we interrogated the requirement for NOD2 signaling in (MDP + LPS)-induced IL-1β synergy using the NOD2 inhibitor GSK717. SHIP+/+ and SHIP−/− MCSF-derived BMDMs were pretreated with vehicle control (0.1% DMSO) or GSK717 for 30 min, and then stimulated with MDP, LPS, or MDP + LPS. ATP was added during the final hour of stimulation. In SHIP+/+ BMDMs, MDP + LPS stimulation produced higher IL-1β concentrations than LPS, although the difference was not statistically significant, and GSK717 only modestly reduced IL-1β (Fig. 8). As in previous experiments, SHIP−/− BMDMs produced significantly more IL-1β in response to MDP + LPS, and synergy was blocked by GSK717. IL-6 and TNFα also showed slight increases with MDP + LPS stimulation compared to LPS alone for both SHIP+/+ and SHIP−/− macrophages, but were unaffected by NOD2 inhibition. GSK717 did not significantly affect *Il1b*, *Il6*, or *Tnf* mRNA expression (Figure S7a), caspase-1 activation (Figure S7b), or cytotoxicity (Figure S8) compared to vehicle control. Together, these results demonstrate that although NOD2 activation alone induces minimal cytokine production, NOD2 signaling is essential for MDP + LPS-induced IL-1β synergy in SHIP−/− macrophages.

### 3.8. MDP primes LPS-induced cytokine production in BMDMs

**Figure 9:**
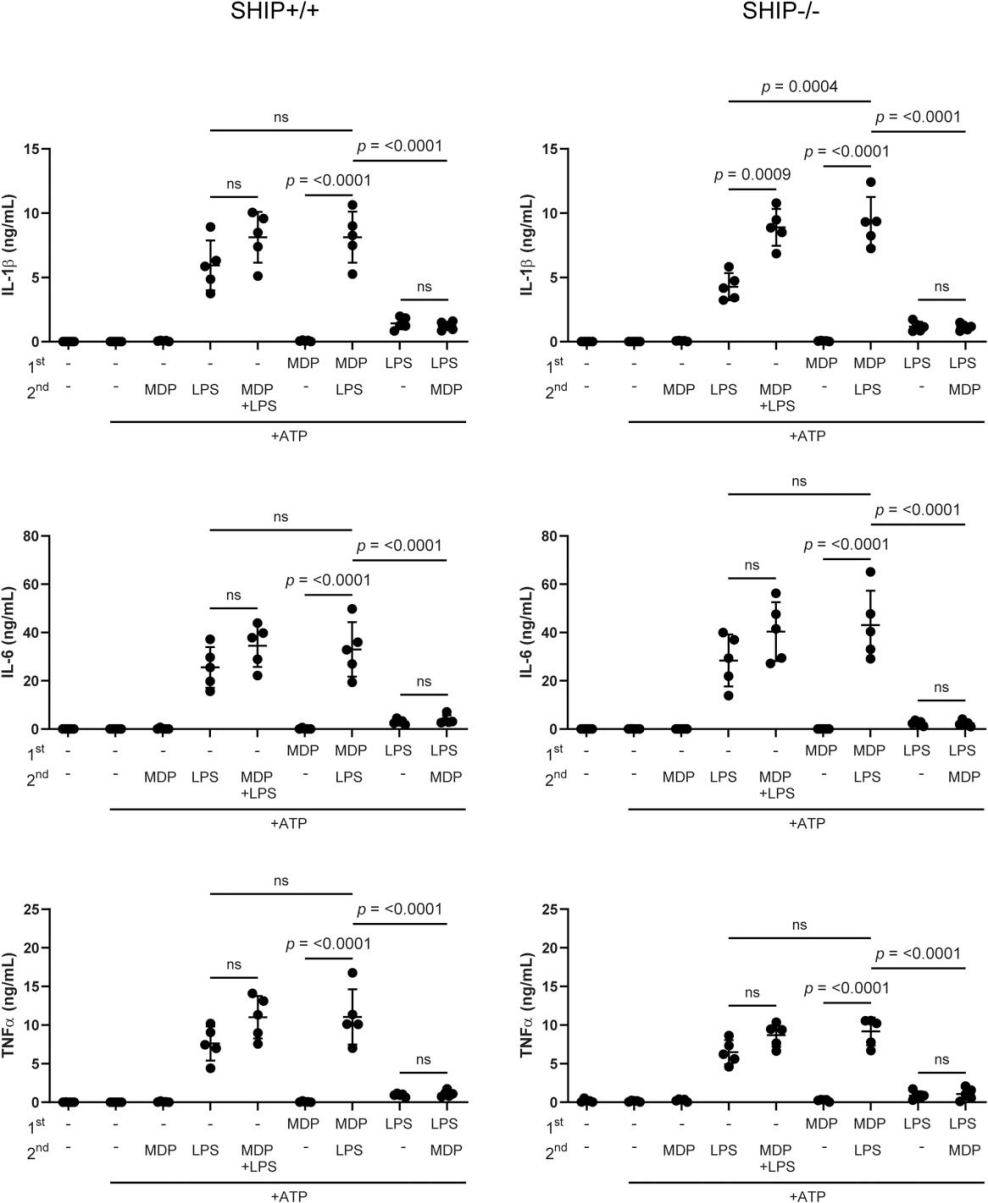
MDP primes BMDMs for increased cytokine responses to LPS. In sequential stimulations, SHIP+/+ and SHIP−/− MCSF-derived BMDMs were untreated or stimulated with MDP (1 µg/mL) or LPS (10 ng/mL) for 24 h. BMDMs were washed with warm PBS, and then stimulated in fresh media ± MDP or LPS for 24 h. ATP (5 mM) was added during the final hour of the 2nd 24 h stimulation. Clarified cell supernatants were assayed by ELISA for IL-1β, IL-6, and TNFα. Data are expressed as mean ± SD for *n* = 5. Statistical analyses were performed using a one-way ANOVA with Sidak's multiple comparisons test. *P* values are stated for comparisons indicated. ns = not statistically significant.

**Figure 10:**
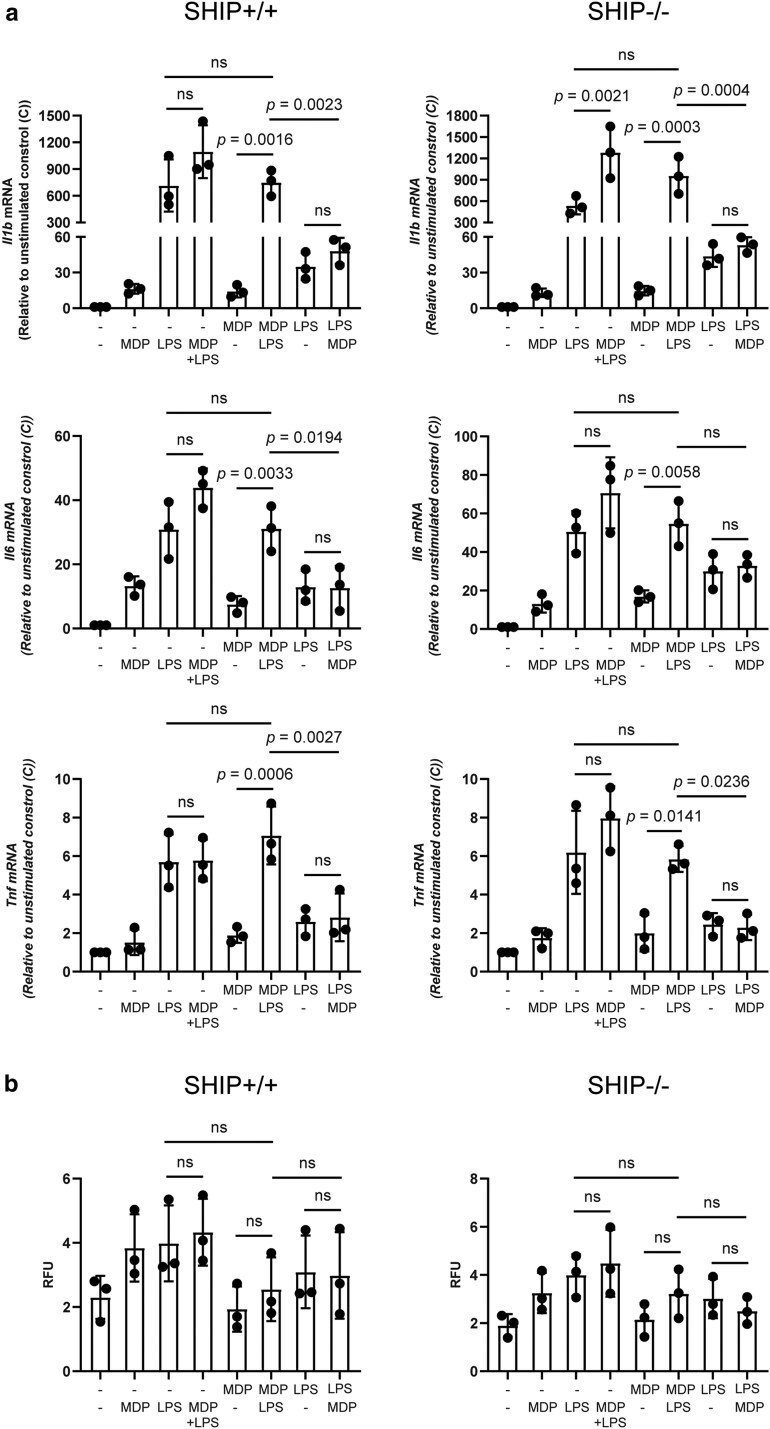
MDP-dependent priming increases *Il1b* expression following subsequent LPS stimulation without increasing caspase-1 activation. In sequential stimulations, SHIP+/+ and SHIP−/− MCSF-derived BMDMs were untreated or stimulated with MDP (1 µg/mL) or LPS (10 ng/mL) for 24 h. BMDMs were washed with warm PBS and then stimulated in fresh media with or without MDP or LPS for 24 h. a) *Il1b*, *Il6*, and *Tnf* expression were measured by RT-qPCR, normalized to *Gapdh*, and reported relative to unstimulated control. b) Active caspase-1 was measured using FAM-FLICA staining and quantified as relative fluorescence units (RFU). Data are expressed as mean ± SD for *n* = 3. Statistical analyses were performed using a one-way ANOVA with Sidak's multiple comparisons test. *P* values are stated for comparisons indicated. ns = not statistically significant.

To determine whether the order of NOD2 and TLR4 stimulation is important for synergistic IL-1β production and how this is regulated by SHIP, we performed sequential macrophage stimulation experiments. MCSF-derived SHIP+/+ and SHIP−/− BMDMs were treated with MDP or LPS for 24 h, washed, and incubated for another 24 h either in fresh medium alone or with the alternate ligand. Single-ligand and simultaneous MDP + LPS controls were added only during the second 24 h period, and ATP was added during the final hour before supernatant collection. For IL-1β, both SHIP+/+ and SHIP−/− BMDMs produced more cytokine when MDP preceded LPS, and these responses were similar to MDP + LPS co-stimulation (Fig. 9). However, synergy remained restricted to SHIP−/− BMDMs, as MDP pretreatment in SHIP+/+ cells did not significantly enhance IL-1β compared to LPS alone. In contrast, LPS priming followed by MDP stimulation did not increase IL-1β production in either genotype. IL-6 and TNFα followed a similar order-dependent pattern in both SHIP+/+ and SHIP−/− BMDMs. Cytokine responses were modestly increased when cells were primed with MDP, and resulted in concentrations similar to those observed with MDP + LPS co-stimulation. However, neither MDP priming followed by LPS nor MDP + LPS co-stimulation produced significantly more IL-6 or TNFα than LPS alone. *Il1b*, *Il6,* and *Tnf* mRNA expression were measured under the same sequential stimulation conditions. Similar to IL-1β production, *Il1b* expression was higher when MDP pretreatment was followed by LPS stimulation than when the order of stimulation was reversed in both SHIP+/+ and SHIP−/− BMDMs (Fig. 10a). MDP pretreatment followed by LPS also increased *Il1b* expression compared to MDP pretreatment alone in both genotypes. *Il6* and *Tnf* expression were higher when MDP pretreatment was followed by LPS stimulation in SHIP+/+ BMDMs, whereas in SHIP−/− BMDMs, this response was significant for *Tnf* but not *Il6*. These findings suggest that prior MDP stimulation increases the cytokine mRNA response to subsequent LPS stimulation, supporting a transcriptional contribution to the increased IL-1β production observed. Caspase-1 activation was also measured, and MDP pretreatment followed by LPS stimulation did not increase caspase-1 activation compared to the reverse stimulation order or simultaneous MDP + LPS co-stimulation (Fig. 10b). Taken together, these results suggest that SHIP prevents MDP-dependent priming that enables synergy for IL-1β production in response to subsequent LPS stimulation.

## 4. Discussion

Our findings identify SHIP as a negative regulator of cytokine responses during NOD2–TLR4 co-stimulation and are consistent with an adaptor role for SHIP that is independent of its catalytic activity. SHIP−/− BMDMs show a synergistic increase in IL-1β upon co-stimulation with MDP + LPS, whereas SHIP+/+ cells do not. Synergy for IL-1β production occurs when SHIP protein concentrations are reduced, whether by macrophage differentiation in GM-CSF or IL-3, activation by IL-4 treatment, or SHIP-targeting siRNAs. This response does not depend on SHIP's catalytic activity or PI3K signaling, indicating a nonenzymatic mechanism of regulation. Accordingly, inhibition of NOD2 selectively reduced IL-1β synergy in SHIP−/− BMDMs. Synergy was observed during co-stimulation or when MDP stimulation preceded LPS, but reversing the order of stimulation failed to enhance IL-1β production. These findings support a model in which SHIP acts via its adaptor function to negatively regulate IL-1β production during NOD2 and TLR4 co-stimulation.

Across the macrophage populations in which MDP + LPS enhanced IL-1β production, including MCSF-derived SHIP−/− BMDMs, GM-CSF- and IL-3-derived BMDMs, IL-4-treated BMDMs, and SHIP knockdown BMDMs, this response was associated with an increase in *Il1b* expression without an increase in caspase-1 activation. Since caspase-1 mediates inflammasome-dependent pro-IL-1β processing, the absence of increased caspase-1 activation argues against enhanced inflammasome activation as the primary explanation for increased IL-1β production. Instead, the strong association with *Il1b* expression suggests that SHIP primarily limits the transcriptional priming component of IL-1β production during NOD2 and TLR4 co-stimulation. However, regulation at the level of *Il1b* mRNA stability, translation, pro-IL-1β protein accumulation, or post-translational modification cannot be excluded. Cytotoxicity, assessed by LDH release, was not increased by MDP + LPS co-stimulation, indicating that increased IL-1β production was not explained by increased cell death under these conditions.

BMDMs differentiated under different growth factors show distinct baseline phenotypes and cytokine responsiveness. MCSF-derived macrophages are generally described as homeostatic populations with lower expression of activation markers and reduced inflammatory cytokine production upon stimulation.^56,57^ Compared to GM-CSF-derived macrophages, MCSF-derived cells have lower expression of MHCII, CD11c, and CD80, suggesting limited antigen-presenting capacity.^56,58^ Under inflammatory conditions, GM-CSF is produced by activated immune and stromal cells, in contrast to the constitutive expression of MCSF.^56,59,60^ GM-CSF drives the differentiation of macrophages with an inflammatory phenotype characterized by enhanced responsiveness to microbial ligands, increased secretion of IL-1β, IL-6, TNFα, and nitric oxide following TLR stimulation, and reduced phagocytic capacity compared to MCSF-derived macrophages^57,61–63^. In addition to these differences, GM-CSF-derived macrophages have been reported to express less SHIP than MCSF-derived populations.^48^ This differentiation environment therefore primes macrophages toward an activated state that is more permissive for cytokine production and for synergy following PRR co-stimulation. IL-3 also supports macrophage differentiation and generates populations distinct from those produced with MCSF or GM-CSF. Compared with MCSF- and GM-CSF-derived BMDMs, IL-3-derived BMDMs from BALB/c mice produce higher amounts of prostaglandin E_2_, reflecting differences in inflammatory mediator production.^64^ Short-term IL-3 exposure has also been shown to prime human monocytes for stronger responses to microbial ligands by increasing LPS-induced TNFα production via p38− and SIRT2-dependent post-transcriptional regulation.^65^ Differentiation in GM-CSF or IL-3 produces macrophages with features associated with stronger responses to microbial ligands, including reduced SHIP protein, which is consistent with the increased IL-1β production observed upon co-stimulation with MDP and LPS. The responses of SHIP−/− BMDMs across the same differentiation conditions help distinguish effects linked to SHIP loss from those shaped by macrophage differentiation state. In this context, IL-1β production remains increased by MDP + LPS across MCSF-, GM-CSF-, and IL-3-derived SHIP−/− BMDMs. In contrast, increased IL-6 production remains restricted to GM-CSF- and IL-3-derived SHIP−/− BMDMs, and TNFα production is not increased by MDP + LPS in any of these populations. These observations support a selective role for SHIP in limiting IL-1β production, while reinforcing that macrophage differentiation state also shapes cytokine responsiveness to combined NOD2 and TLR4 stimulation.

Altered expression of receptors involved in MDP or LPS sensing could also contribute to the responsiveness of GM-CSF- and IL-3-derived BMDMs. LPS signaling through TLR4 increased expression of *Nod2*, which may contribute to (MDP + LPS)-induced synergy for IL-1β production. However, experiments examining the effect of sequential activation of NOD2 and TLR4 demonstrate that synergy is only achieved when NOD2 stimulation precedes TLR4 stimulation arguing against a role for LPS-induced upregulation of *Nod2* as a key mechanism required for the synergistic IL-1β response. Moreover, MDP stimulation did not affect *Tlr4* expression, which suggests that synergy is independent of effects on receptor expression. Synergy may be dependent on receptor availability, localization, ligand uptake, or downstream signaling complex formation, all of which may contribute to differential responsiveness to co-stimulation.

IL-4 is the canonical “M2” polarizing cytokine that drives M(IL-4) differentiation.^66^ M(IL-4) macrophages are generally considered less inflammatory than classically activated macrophages, and produce relatively lower amounts of proinflammatory cytokines.^67^ In human monocytes, IL-4 suppresses LPS-induced TNFα, and in a separate study IL-4 co-treatment also reduced LPS-induced TNFα, IL-1, and PGE_2_.^68,69^ In conventional dendritic cells, IL-4 suppresses TNFα, IL-12p70, and IL-6 in response to TLR7 and TLR9 agonists by inhibiting interferon signaling and NFκB pathways.^70^ These studies support the widely held view that IL-4 limits proinflammatory cytokine production. However, this pattern is not universal, and there are contexts in which IL-4 enhances inflammatory responses rather than limiting them. In thioglycollate-elicited mouse peritoneal macrophages, IL-4 pretreatment amplifies LPS-induced TNFα, IL-1α, MIP2, and KC 2− to 4-fold in a STAT6-dependent manner.^71^ Moreover, IL-4-treated macrophages can retain an M(IL-4) transcriptional profile yet become more responsive to subsequent LPS stimulation, and this heightened responsiveness depends on glycolysis and HIF-1α activity.^72^ Recent work shows that IL-4 broadly remodels macrophage responses across multiple TLRs, including TLR1/2, TLR3, TLR4, TLR5, TLR2/6, TLR7/8, and TLR9, by expanding the NFκB cistrome and increasing enhancer activity.^73^ Although the study did not examine NOD2, it demonstrates that IL-4 broadens NFκB-dependent chromatin accessibility across multiple TLRs, and provides a plausible mechanism for higher cytokine production when more than one receptor is activated.

Within this framework, our data shows that IL-4 has distinct effects on single ligand versus combined stimulation. Consistent with expectations that M(IL-4) macrophages are less inflammatory, our M(IL-4) macrophages produce less IL-6 and TNFα in response to LPS than LPS-stimulated BMDMs that are not exposed to IL-4. Unexpectedly, the same IL-4 pretreatment increases IL-1β, IL-6, and TNFα production when cells are stimulated with MDP + LPS. In this model, IL-4 pretreatment reduces SHIP protein in MCSF-derived BMDMs, which is consistent with previous work demonstrating that IL-4 promotes proteasomal degradation of SHIP in macrophages.^48^ Reduced SHIP abundance may contribute to the IL-1β component of this response, since SHIP knockdown selectively enhances IL-1β production during MDP + LPS co-stimulation. However, IL-4-treated SHIP−/− BMDMs also show increased IL-1β, IL-6, and TNFα production following MDP + LPS co-stimulation compared to LPS alone. This indicates that IL-4-dependent enhancement of cytokine responses may not be simply attributed to reduced SHIP protein concentration, and that broader IL-4-driven changes in macrophage activation state may also contribute to responsiveness to combined NOD2 and TLR4 stimulation. The suppression of LPS-only responses despite increased synergy with MDP + LPS may reflect differential metabolic or chromatin remodeling thresholds that are only overcome during co-stimulation. Together, these observations reinforce the idea that IL-4 does not uniformly dampen inflammatory signaling but instead reshapes the inflammatory landscape in a receptor- and context-specific manner.

Reducing SHIP expression by siRNA in MCSF-derived BMDMs allowed us to assess the specific contribution of SHIP to MDP + LPS synergy without introducing changes to macrophage differentiation or activation. SHIP knockdown is sufficient to increase IL-1β production in response to MDP + LPS co-stimulation, and is consistent with observations in GM-CSF- and IL-3-differentiated, and IL-4-activated macrophages. This response is accompanied by a selective increase in *Il1b* expression without an increase in caspase-1 activation. In contrast, IL-6 and TNFα production are not enhanced by SHIP knockdown, suggesting that the effect is not a general amplification of cytokine responses but rather reflects a more selective role for SHIP in regulating IL-1β production. These findings help clarify that SHIP concentrations directly influence the threshold for IL-1β synergy during NOD2 and TLR4 co-stimulation, independent of broader transcriptional or metabolic shifts associated with differentiation or activation.

Pharmacologic inhibition of SHIP phosphatase activity with 3AC does not reproduce the enhanced cytokine production observed when SHIP protein concentration is low. In iPSC-derived human microglia, 3AC treatment increased the secretion of IL-1β and IL-18 through NLRP3–caspase-1 inflammasome activation, indicating that inhibition of SHIP activity can enhance inflammasome-dependent cytokine production in myeloid cells.^74^ Trained immunity models using mouse BMDMs and human PBMCs have similarly shown that 3AC increases LPS-induced TNFα production following prior β-glucan exposure in BMDMs, and increases IL-1β, IL-6, and TNFα upon LPS rechallenge in PBMCs.^75^ In *Leishmania* infection, peritoneal macrophages infected with *L. donovani* and treated with 3AC showed increased IL-12p70, TNFα, and IFNγ, reduced IL-10 and TGFβ, and a decreased parasite burden.^76^ Collectively, these reports establish that inhibition of SHIP's phosphatase activity by 3AC can increase cytokine concentrations in other models, but in a model of NOD2–TLR4 co-stimulation of MCSF-derived BMDMs, 3AC does not increase IL-1β, IL-6, or TNFα in SHIP+/+ cells, indicating that the synergy observed is independent of SHIP's phosphatase activity. Consistent with a phosphatase-independent role for SHIP, blocking PI3K does not reduce the increased IL-1β production observed after NOD2–TLR4 co-stimulation. In SHIP−/− BMDMs, LY294002 reduces IL-1β, IL-6, and TNFα, but the same reduction with the inactive analog LY303511, and the lack of an effect with wortmannin argues against a class I PI3K mechanism. Reports from multiple groups show that LY294002 and LY303511 share PI3K-independent activities that can reduce cytokine production, including inhibition of BET bromodomain proteins, casein kinase 2, NO production, iNOS expression, and NFκB DNA binding and reporter activity^77–81^. Taken together, these findings indicate that the increased IL-1β production observed after MDP + LPS co-stimulation in SHIP−/− MCSF-derived BMDMs is independent of PI3K activity and SHIP's phosphatase activity. Blocking NOD2 with GSK717 abolishes MDP + LPS synergy for IL-1β production in SHIP−/− BMDMs. IL-6 and TNFα were unchanged, reflecting the selective dependence of IL-1β synergy on NOD2 in the absence of SHIP. Across the pharmacologic treatment conditions, LDH release was not increased by 3AC, LY303511, LY294002, wortmannin, or GSK717, making cytotoxicity unlikely to be a major confounder in interpreting the cytokine responses to these inhibitors.

The requirement for NOD2 aligns with sequential stimulation studies showing that prior MDP exposure increases the subsequent LPS response in THP-1 cells, an effect lost in THP-1 NOD2-deficient cells, and in primary NOD2-deficient bone-marrow monocytes.^82^ THP-1 cells differentiated with 22-oxyacalcitriol show a similar direction of effect, with MDP given first augmenting the later LPS response to produce high concentrations of IL-8, whereas the reverse order has little impact.^28^ Published models suggest that NOD2 can promote later TLR4 responses through multiple mechanisms, including formation of a NALP1-caspase-1 complex that enables caspase-1-dependent maturation of pro-IL-1β, and early autocrine IL-1β release downstream of NOD2 that amplifies MAPK signaling during subsequent TLR4 stimulation.^83,84^ In SHIP+/+ and SHIP−/− BMDMs, the order-dependent IL-1β response appears to be more closely associated with *Il1b* induction, since *Il1b* expression is higher when MDP precedes LPS than when the order of stimulation is reversed, while caspase-1 activation is not increased compared to the reverse order or simultaneous co-stimulation. Reduced SHIP concentrations may further favor this transcriptional bias toward IL-1β production, reflected by higher LPS-induced *Il1b* transcription via PI3K p110α in SHIP−/− peritoneal macrophages, and by an inverse relationship between SHIP activity and inducible IL-1β in human PBMCs.^49^

## 5. Conclusion

SHIP limits IL-1β production during MDP + LPS co-stimulation through a noncatalytic mechanism linked to SHIP protein abundance, consistent with its role as an adaptor. Enhanced IL-1β production is associated with increased *Il1b* expression rather than increased caspase-1 activation. These findings demonstrate the complexity of targeting innate immune pathways in macrophages, where receptor interactions and differentiation environments shape the magnitude and specificity of cytokine responses. Although SHIP limits IL-1β production independently of catalytic activity, modulating SHIP concentrations is not easily achievable in vivo, and the tissue-specific consequences of altering macrophage activation states will vary in complex and context-dependent environments. Using reductionist approaches to examine SHIP's role in macrophages in multiple differentiation and activation conditions, SHIP emerges as a gatekeeper for macrophage IL-1β production. Future studies should explore how SHIP abundance is regulated during inflammation and whether SHIP scaffolding or adaptor functions intersect with signaling pathways beyond NOD2 and TLR4. A deeper understanding of how PRR synergy is regulated may help identify novel therapeutic strategies to limit pathological IL-1β production in inflammatory diseases including IL-1β-mediated pyrinopathies.

## Supplementary Material

qiag090_Supplementary_Data

## Data Availability

Data will be made available upon reasonable request.
