# Supplementary material for: SHIP blocks (MDP + LPS)-induced synergy in macrophages independent of catalytic activity
Source: J Leukoc Biol. 2026 Jun 27;118(7):qiag090. doi: 10.1093/jleuko/qiag090 (PMC13344797; doi:10.1093/jleuko/qiag090)
Supplement: qiag090_Supplementary_Data [file qiag090_supplementary_data.pdf]

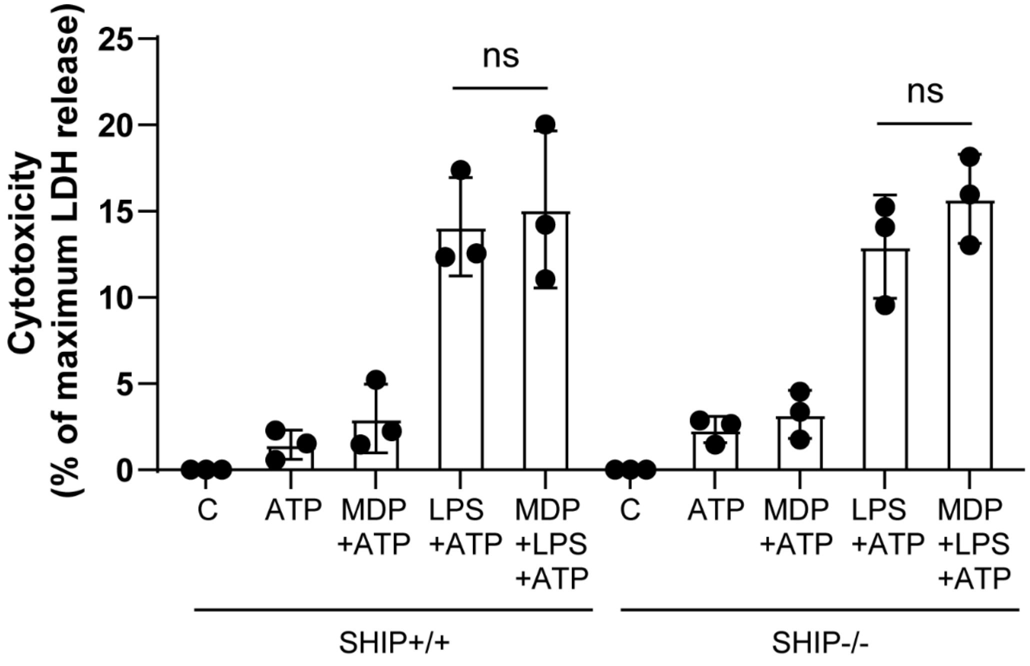

Figure S1. Cytotoxicity is not increased by MDP+LPS co-stimulation. Cytotoxicity was assessed by LDH release and expressed as percent of maximum LDH release. MCSF-derived SHIP+/+ and SHIP-/- BMDMs were stimulated with MDP (1 μg/mL), LPS (10 ng/mL), or MDP+LPS for 24 h. ATP (5 mM) was added for the final hour. Data are expressed as mean ± SD for n = 3. Statistical analyses were performed using a one-way ANOVA with Sidak's multiple comparisons test. P values are stated for comparisons indicated. ns = not statistically significant.

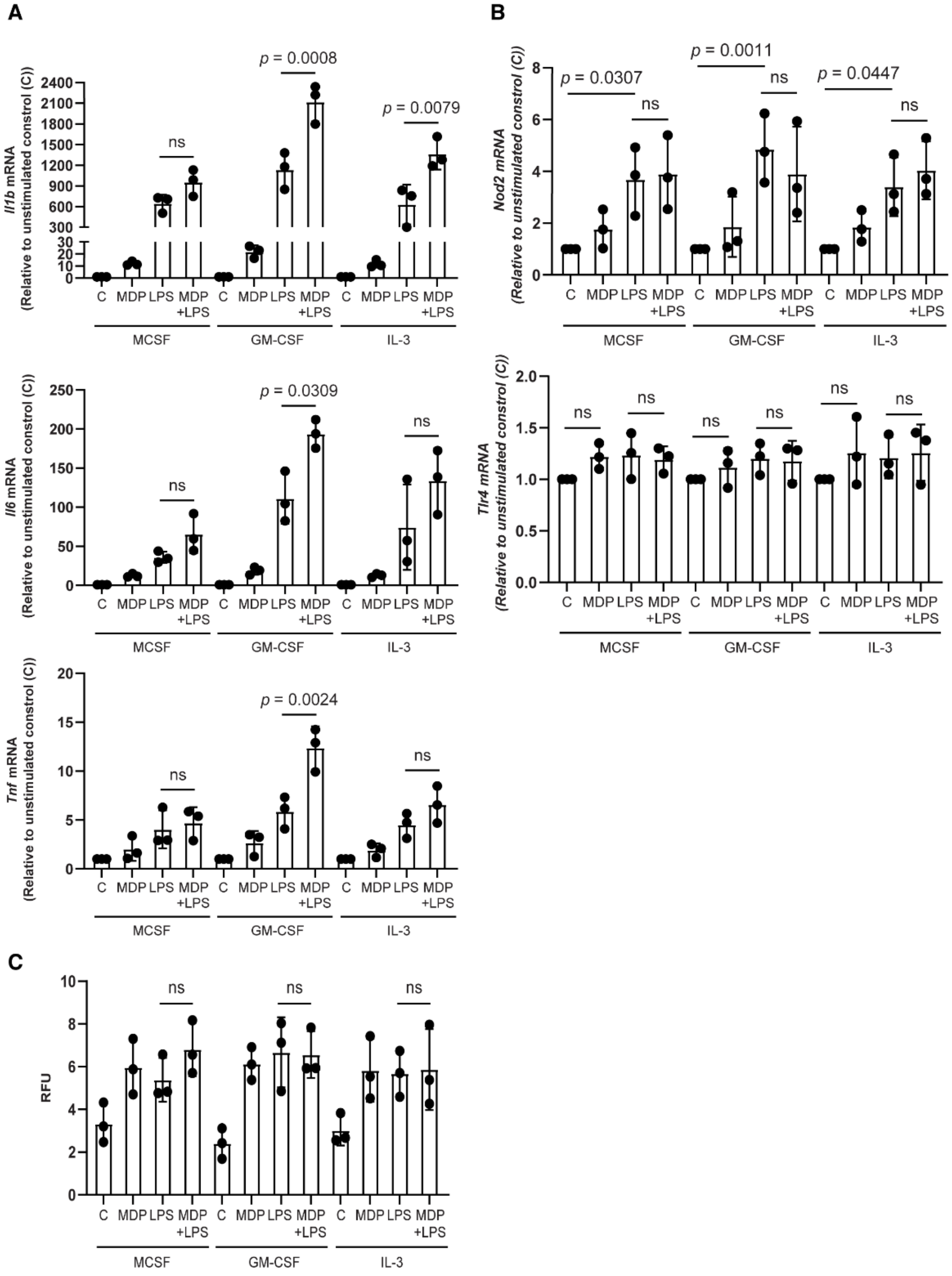

Figure S2. MDP+LPS co-stimulation increases *Il1b* expression in GM-CSF- and IL-3-derived SHIP+/+ BMDMs. MCSF-, GM-CSF-, and IL-3-derived SHIP+/+ BMDMs were stimulated with MDP (1 µg/mL), LPS (10 ng/mL), or MDP+LPS for 24 h. (A) *Il1b*, *Il6*, *Tnf*, *Nod2*, and *Tlr4* expression were measured by RT-qPCR, normalized to *Gapdh*, and reported relative to unstimulated control. (B) Active caspase-1 was measured using FAM-FLICA staining and quantified as relative fluorescence units (RFU). Data are expressed as mean ± SD for n = 3. Statistical analyses were performed using a one-way ANOVA with Sidak's multiple comparisons test. P values are stated for comparisons indicated. ns = not statistically significant.

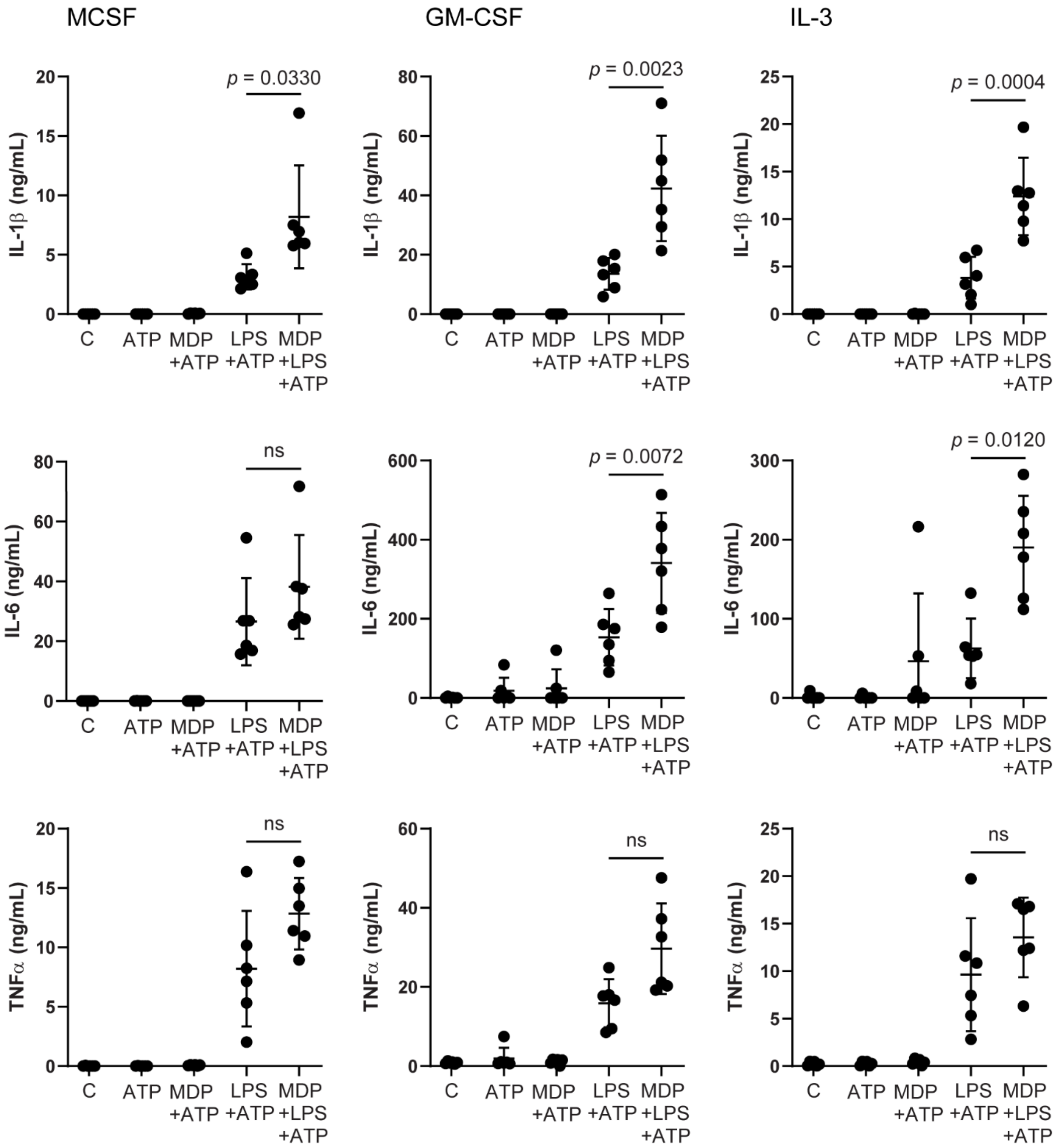

Figure S3. MDP+LPS co-stimulation increases IL-1β production in SHIP-/- BMDMs derived with MCSF, GM-CSF, or IL-3. MCSF-, GM-CSF-, and IL-3-derived SHIP-/- BMDMs were stimulated with MDP (1 µg/mL), LPS (10 ng/mL), or MDP+LPS for 24 h. ATP (5 mM) was added for the final hour. Clarified cell culture supernatants were assayed by ELISA for IL-1β, IL-6, and TNFα. Data are expressed as mean ± SD for n = 6. Statistical analyses were performed using a one-way ANOVA with Sidak's multiple comparisons test. P values are stated for comparisons indicated. ns = not statistically significant.

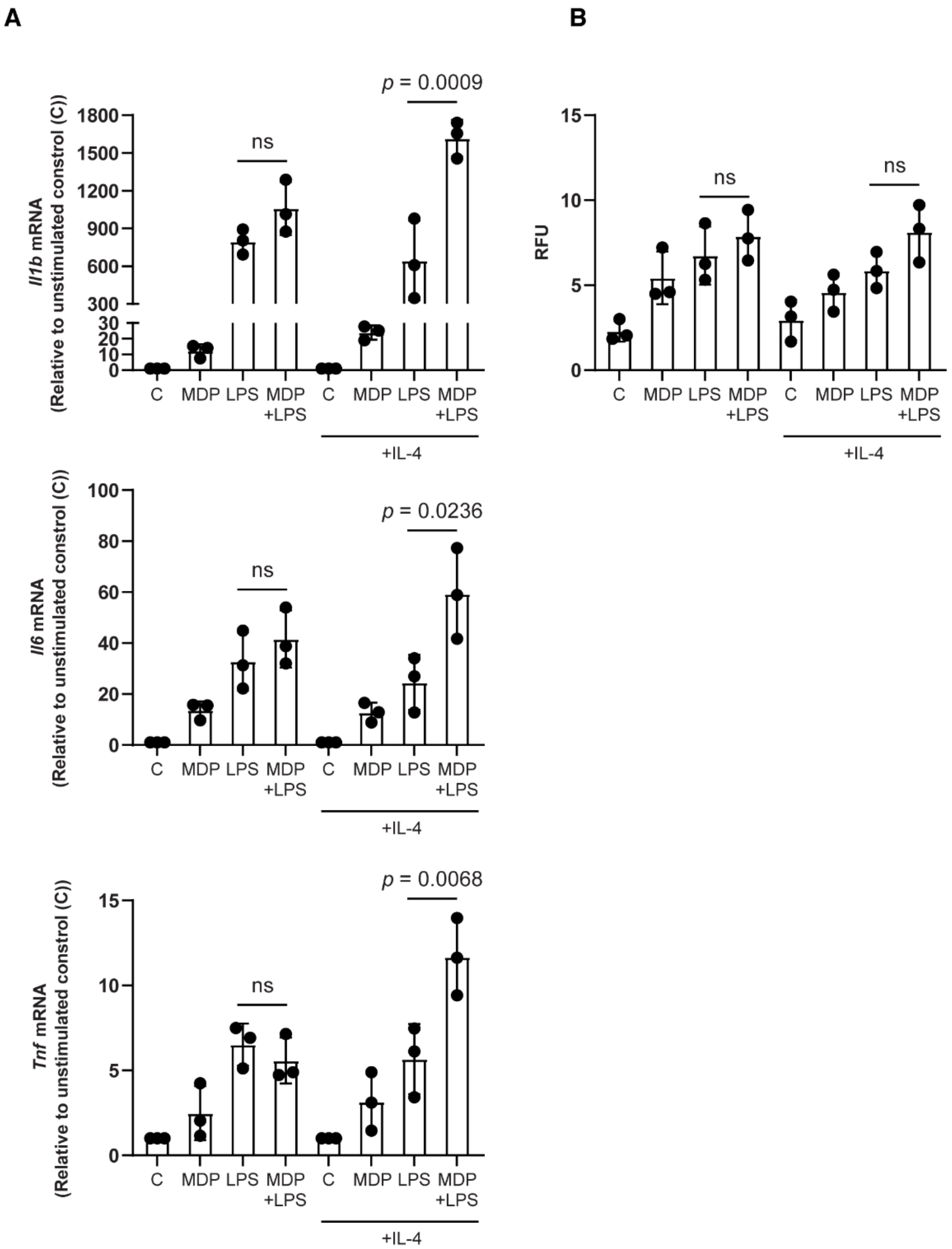

Figure S4. IL-4 treatment increases (MDP+LPS)-induced *Il1b*, *Il6*, and *Tnf* expression in SHIP+/+ MCSF-derived BMDMs. SHIP+/+ MCSF-derived BMDMs were cultured with or without IL-4 (10 ng/mL) for 72 h and then stimulated with MDP (1 µg/mL), LPS (10 ng/mL), or MDP+LPS for 24 h. (A) *Il1b*, *Il6*, and *Tnf* expression were measured by RT-qPCR, normalized to *Gapdh*, and reported relative to unstimulated control. (B) Active caspase-1 was measured using FAM-FLICA staining and quantified as relative fluorescence units (RFU). Data are expressed as mean ± SD for n = 3. Statistical analyses were performed using a one-way ANOVA with Sidak's multiple comparisons test. P values are stated for comparisons indicated. ns = not statistically significant.

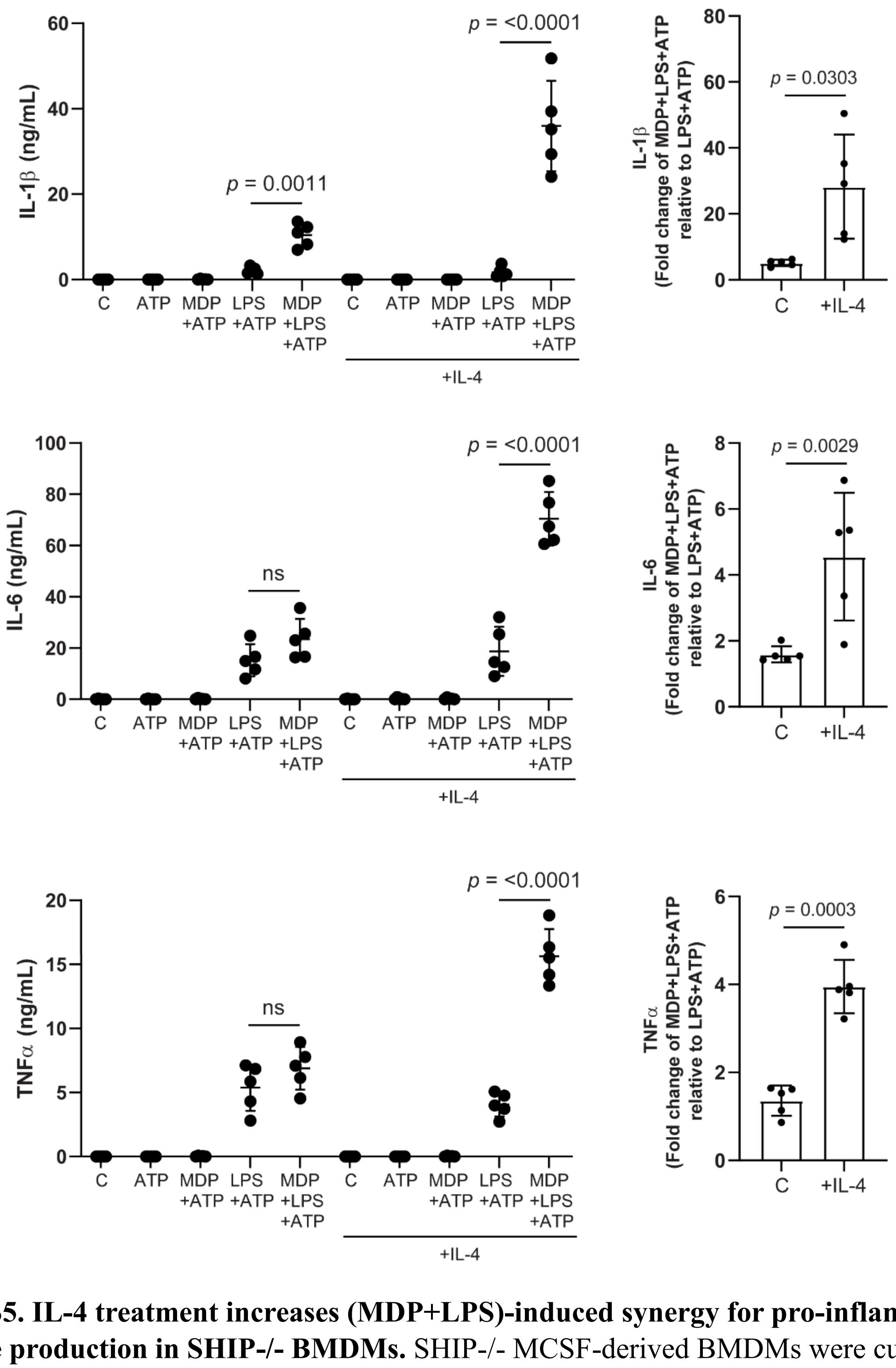

Figure S5. IL-4 treatment increases (MDP+LPS)-induced synergy for pro-inflammatory cytokine production in SHIP-/- BMDMs. SHIP-/- MCSF-derived BMDMs were cultured with or without IL-4 (10 ng/mL) for 72 h and then stimulated with MDP (1 μg/mL), LPS (10 ng/mL), or MDP+LPS for 24 h. ATP (5 mM) was added for the final hour. Clarified cell culture supernatants were assayed by ELISA for IL-1β, IL-6, and TNFα. Data are expressed as mean ± SD for n = 5. Statistical analyses were performed using a one-way ANOVA with Sidak's multiple comparisons test. P values are stated for comparisons indicated. ns = not statistically significant.

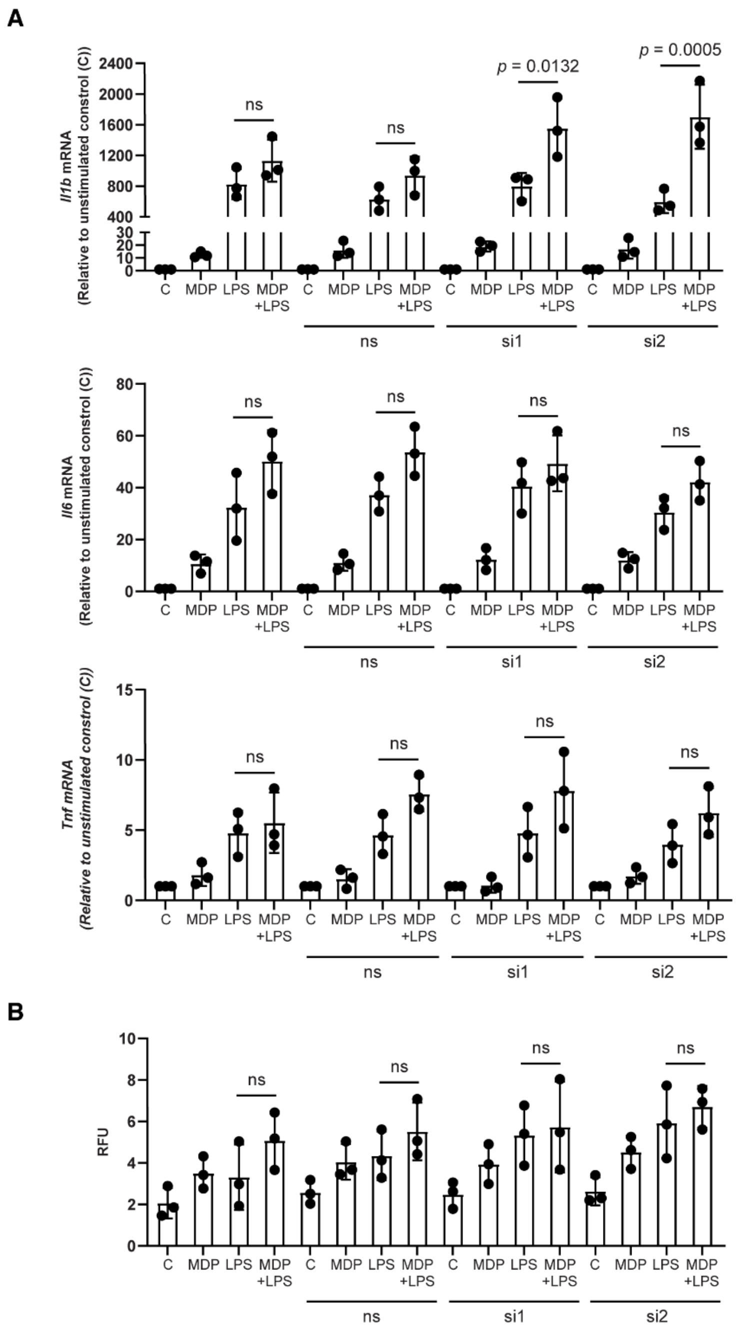

Figure S6. SHIP knockdown increases *Il1b* expression after MDP+LPS stimulation in SHIP+/+ MCSF-derived BMDMs. MCSF-derived SHIP+/+ BMDMs were treated with non-silencing RNA control (ns) or two independent SHIP-targeting siRNAs (si1 and si2) for 72 h, prior to stimulation with MDP (1 µg/mL), LPS (10 ng/mL), or MDP+LPS for 24 h. (A) *Il1b*, *Il6*, and *Tnf* expression were measured by RT-qPCR, normalized to *Gapdh*, and reported relative to unstimulated control. (B) Active caspase-1 was measured using FAM-FLICA staining and quantified as relative fluorescence units (RFU). Data are expressed as mean ± SD for n = 3. Statistical analyses were performed using a one-way ANOVA with Sidak's multiple comparisons test. P values are stated for comparisons indicated. ns = not statistically significant.

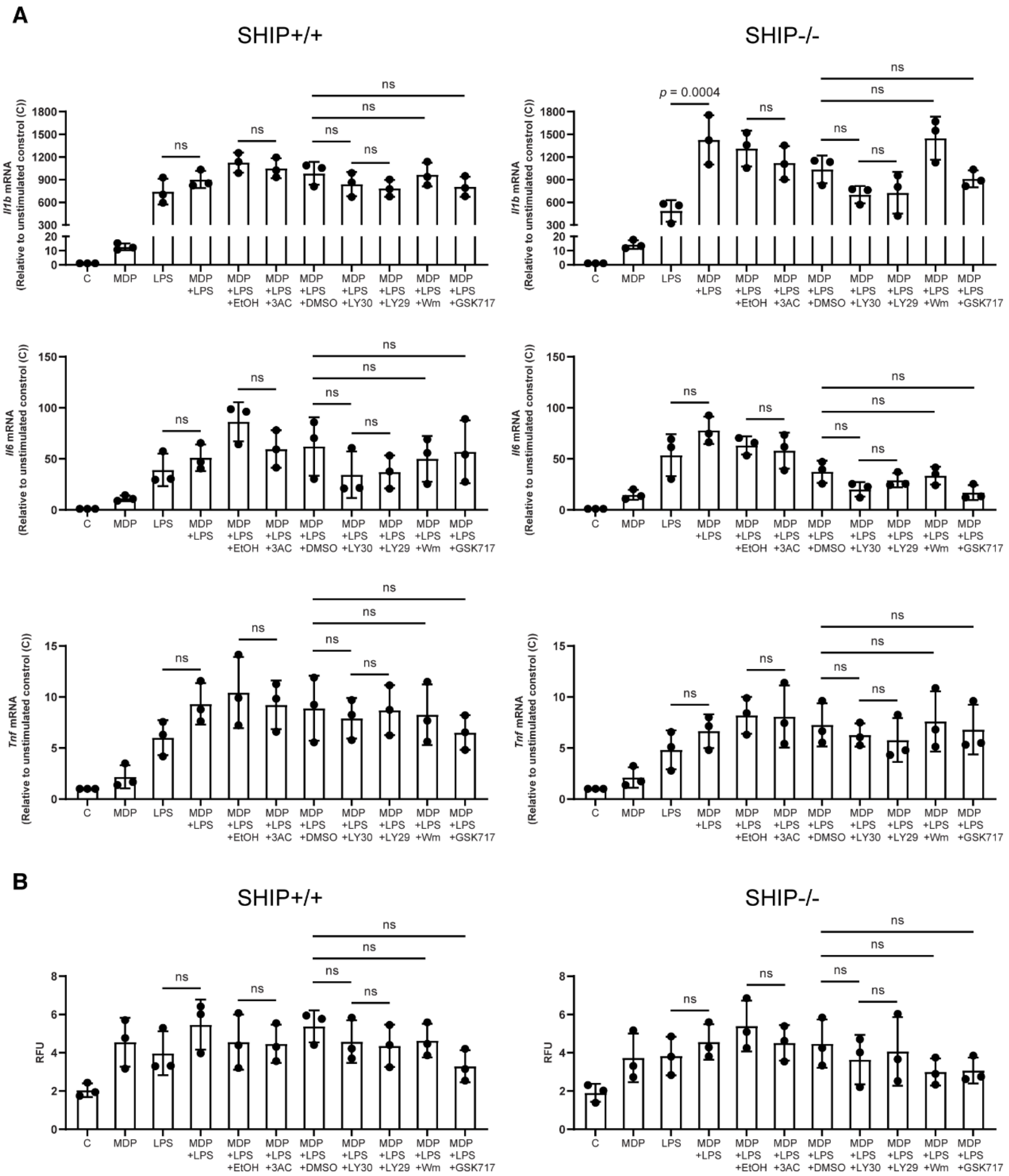

Figure S7. 3AC, LY30, LY29, Wm, and GSK717 did not significantly alter *Il1b*, *Il6*, or *Tnf* expression, or caspase-1 activation. SHIP+/+ and SHIP-/- MCSF-derived BMDMs were pre-treated for 30 min with vehicle control, 3AC (20 µM), LY30 (10 µM), LY29 (10 µM), Wm (100 nM), or GSK717 (10 µM), and then stimulated with MDP (1 µg/mL), LPS (10 ng/mL), or MDP+LPS for 24 h. (A) *Il1b*, *Il6*, and *Tnf* expression were measured by RT-qPCR, normalized to *Gapdh*, and reported relative to unstimulated control. (B) Active caspase-1 was measured using FAM-FLICA staining and quantified as relative fluorescence units (RFU). Data are expressed as mean ± SD for n = 3. Statistical analyses were performed using a one-way ANOVA with Sidak's multiple comparisons test. P values are stated for comparisons indicated. ns = not statistically significant.

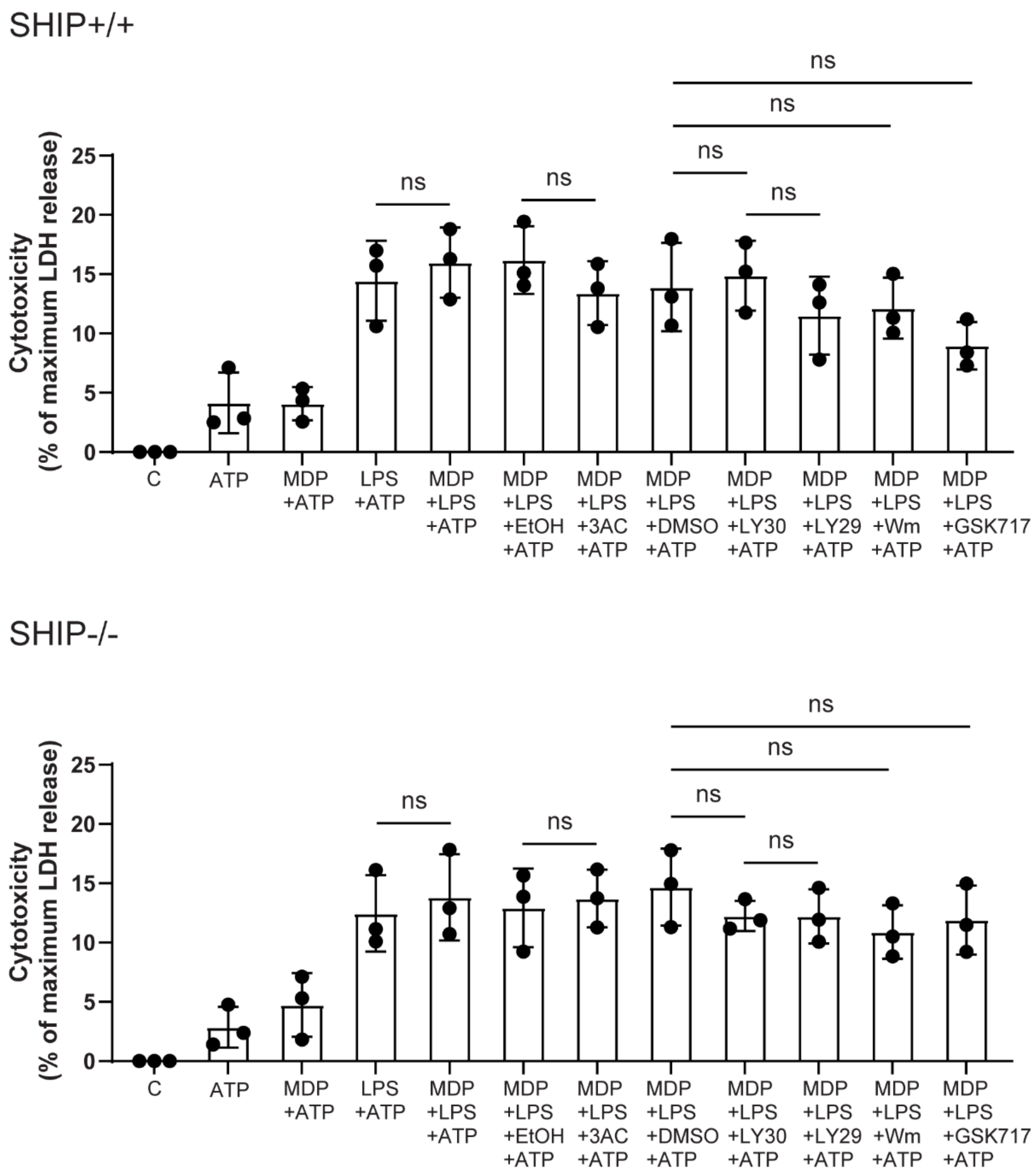

Figure S8. Cytotoxicity is not increased by inhibitor treatment. Cytotoxicity was assessed by LDH release and expressed as percent of maximum LDH release. SHIP+/+ and SHIP-/- MCSF-derived BMDMs were pre-treated for 30 min with vehicle control, 3AC (20 μM), LY30 (10 μM), LY29 (10 μM), Wm (100 nM), or GSK717 (10 μM), and then stimulated with MDP (1 μg/mL), LPS (10 ng/mL), or MDP+LPS for 24 h. ATP (5 mM) was added for the final hour. Data are expressed as mean ± SD for n = 3. Statistical analyses were performed using a one-way ANOVA with Sidak's multiple comparisons test. P values are stated for comparisons indicated. ns = not statistically significant.
